# Multivariate Statistical and Multiproxy Constraints on Earthquake‐Triggered Sediment Remobilization Processes in the Central Japan Trench

**DOI:** 10.1029/2019GC008861

**Published:** 2020-06-18

**Authors:** T. Schwestermann, J. Huang, J. Konzett, A. Kioka, G. Wefer, K. Ikehara, J. Moernaut, T. I. Eglinton, M. Strasser

**Affiliations:** ^1^ Institute of Geology University of Innsbruck Innsbruck Austria; ^2^ Institute of Mineralogy and Petrography University of Innsbruck Innsbruck Austria; ^3^ Department of Earth Resources Engineering Kyushu University Fukuoka Japan; ^4^ MARUM—Center for Marine Environmental Sciences University of Bremen Bremen Germany; ^5^ Geological Survey of Japan National Institute of Advanced Industrial Science and Technology (AIST) Tsukuba Japan; ^6^ Geological Institute ETH Zürich Zürich Switzerland

**Keywords:** surficial sediment remobilization, turbidite, XRF core scanning, paleoseismology, heavy mineral analysis, Japan Trench

## Abstract

Understanding the impact of earthquakes on subaqueous environments is key for submarine paleoseismological investigations seeking to provide long‐term records of past earthquakes. For this purpose, event deposits (e.g., turbidites) are, among others, identified and stratigraphically correlated over broad areas to test for synchronous occurrence of gravity flows. Hence, detailed spatiotemporal petrographic and geochemical fingerprints of such deposits are required to advance the knowledge about sediment source and the underlying remobilization processes induced by past earthquakes. In this study, we develop for the first time in paleoseismology a multivariate statistical approach using X‐ray fluorescence core scanning, magnetic susceptibility, and wet bulk density data that allow to test, confirm, and enhance the previous visual and lithostratigraphic correlation across two isolated basins in the central Japan Trench. The statistical correlation is further confirmed by petrographic heavy grain analysis of the turbidites and additionally combined with our novel erosion model based on previously reported bulk organic carbon ^14^C dates. We find surficial sediment remobilization, a process whereby strong seismic shaking remobilizes the uppermost few centimeters of surficial slope sediment, to be a predominant remobilization process, which partly initiates deeper sediment remobilization downslope during strong earthquakes at the Japan Trench. These findings shed new light on source‐to‐sink transport processes in hadal trenches during earthquakes and help to assess the completeness of the turbidite paleoseismic record. Our results further suggest that shallow‐buried tephra on the slope might significantly influence sediment remobilization and the geochemical and petrographic fingerprints of the resulting event deposits.

## Key Points

- Multivariate statistics on XRF‐CS and MSCL data enhance the correlation of earthquake‐triggered event deposits in the Japan Trench
- Each event deposit reveals a characteristic geochemical, physical, and petrographic fingerprint
- Bulk organic carbon ^14^C and the characteristic fingerprints of event deposits suggest predominantly surficial slope sediment remobilization

## 1 Introduction

Ever since the Grand Banks earthquake in 1929, it has been known that ground shaking can trigger submarine slope failures (e.g., slumps and slides), evolving downslope into debris flows and turbidity currents (Heezen & Ewing, 1952) and depositing in terminal basins as event deposits (e.g., mass‐transport deposits and turbidites). The resulting event deposits retrieved from sedimentary archives have been studied, dated, and correlated, in the research field of “submarine paleoseismology” (De Batist et al., 2017; Goldfinger, 2009; Gràcia et al., 2013; and references therein), to reconstruct magnitudes and recurrence patterns of past earthquakes. This concept has been widely applied in many active margins around the world and provides valuable information for seismic hazard assessments, such as the studies in Cascadia subduction zone (Goldfinger et al., 2017; Gutiérrez‐Pastor et al., 2013), Sumatra‐Andaman subduction zone (Patton et al., 2015), Japan Trench (Ikehara et al., 2016), and Hikurangi margin (Pouderoux et al., 2014, 2012). These studies demonstrate that a robust event‐stratigraphic correlation is fundamental to capture an accurate seismic history. Besides correlating distinct time horizons within the stratigraphic record (e.g., tephra layers) and establishing a precise chronology (e.g., by tephra stratigraphy and radiocarbon dating), many other approaches using sedimentological, petrographical, geochemical, geophysical, and micropaleontological proxies have been proposed to establish a stratigraphic framework and to identify the sedimentologic characteristics and provenances of event deposits (e.g., Beckers et al., 2017; Çağatay et al., 2012; Goldfinger, 2009; Goldfinger et al., 2017, 2012; McHugh et al., 2014; Polonia et al., 2013; Usami et al., 2017). Such “fingerprinting” information provides the spatial and temporal correlations of turbidite records that allow testing for event synchronicity and eventually inferring source and seismic ground motion characteristics of the areas from where sediment has been remobilized. However, most of these methods are time consuming and have relatively low sampling resolution (dm‐ to cm‐level) when considering detailed variations within a single event deposit and in between different event deposits throughout the entire sedimentary system. Therefore, a comprehensive, high‐resolution, and multiproxy approach is needed to develop a robust statistical basis for deciphering the fingerprint of each event deposit.

High‐resolution X‐ray fluorescence core scanning (XRF‐CS) is widely used to rapidly assess elemental variations in sedimentary archives (Croudace et al., 2019; Croudace & Rothwell, 2015). By combining with other indicators (e.g., grain size, X‐ray diffraction (XRD) mineralogy, organic and inorganic geochemistry, and magnetic susceptibility [MS]) and multivariate statistical approaches, many recent XRF‐CS‐based paleoenvironmental studies have demonstrated usefulness of this approach to quickly and reliably characterize specific target layers in paleoarchives (e.g., Chang et al., 2018; Goff et al., 2018; Lee et al., 2018; Peti et al., 2019). To date, there are many XRF‐CS applications in the studies of submarine paleoseismology (e.g., Beckers et al., 2017; Çağatay et al., 2012; Gràcia et al., 2010; McHugh et al., 2014; Polonia et al., 2013; Yakupoğlu et al., 2019). However, XRF‐CS and other multiproxy data were mainly used to descriptively illustrate the differences between event deposits and background sedimentation. Multivariate statistical approaches on such data have not yet been applied for paleoseismologic studies despite their great potential to reveal the fingerprints of event deposits and provide robust event‐stratigraphic correlation.

On the other hand, detailed fingerprinting analysis does not only improve event deposit correlations to understand the spatial and temporal variabilities of event deposits but also provides valuable information regarding the sediment source and remobilization processes (Barnes et al., 2013; Moernaut et al., 2017). Other than slope failures on continental slopes that remobilize several tens to hundreds of meter thick sedimentary sequences (e.g., Hampton et al., 1996; Masson et al., 2006), several recent studies in the Nankai Trough (Ashi et al., 2014), Chilean lakes (Moernaut et al., 2017), and the Japan Trench (Kioka et al., 2019a; McHugh et al., 2016; Molenaar et al., 2019) reported that earthquakes also trigger surficial sediment remobilization. This remobilization process involves erosion of only the uppermost few centimeters of slope sediments by large seismic shaking over extensive areas (several hundred km^2^). The volumes of remobilized sediments are therefore significant (e.g., ~0.187 km^3^ in the Japan Trench by ad 2011 Tohoku‐oki earthquake, Kioka et al., 2019a; ~0.0024 km^3^ in the Nankai Trough by ad 2004 off‐Kii Peninsula earthquake, Ashi et al., 2014), resulting in meter thick event deposits in depositional basins (e.g., Kioka et al., 2019a).

Such event‐stratigraphic records are expected to reveal complete and continuous paleoseismic records because surficial sediment remobilization is likely to occur over large areas and does not have strict precondition factors compared to massive slides (Moernaut et al., 2017). In the sedimentary record of the depositional basin, this process has so far only been discussed in very young events (<150 years) utilizing short‐lived radionuclides (e.g., ^210^Pb and ^137^Cs, see Kioka et al., 2019a; McHugh et al., 2016; Molenaar et al., 2019). For older event deposits, high‐resolution and statistically robust fingerprinting information combined with longer‐lived radionuclides (e.g., radiocarbon [^14^C] of bulk organic carbon [OC]) are thus needed to better decipher different remobilization processes and test for the significance of surficial sediment remobilization by past earthquakes.

This study aims to examine the temporal and spatial fingerprints of event deposits in the central Japan Trench in order to reveal potential sediment remobilization processes during past, large earthquakes. By using multivariate statistical approaches based on high‐resolution XRF‐CS data combined with MS and gamma‐ray wet bulk density (hereafter: density) data, we reveal the characteristics and stratigraphic correlation of earthquake‐related event deposits over the last 1,500 years. With this information, the provenance of the selected turbidites is further evaluated by heavy mineral analyses with respect to regional background data. Together with the new erosion model based on high‐resolution ^14^C ages of bulk OC throughout the turbidites, the process of surficial sediment remobilization is tested beyond the current limits of the short‐lived radionuclide methods (~150 a). We aim at such process understanding that can shed new light on source‐to‐sink response of hadal trench to great earthquakes at active margins and help to assess the completeness of the turbidite paleoseismic record.

## 2 Study Area and Previous Paleoseismology Studies in the Japan Trench

**Figure 1:**
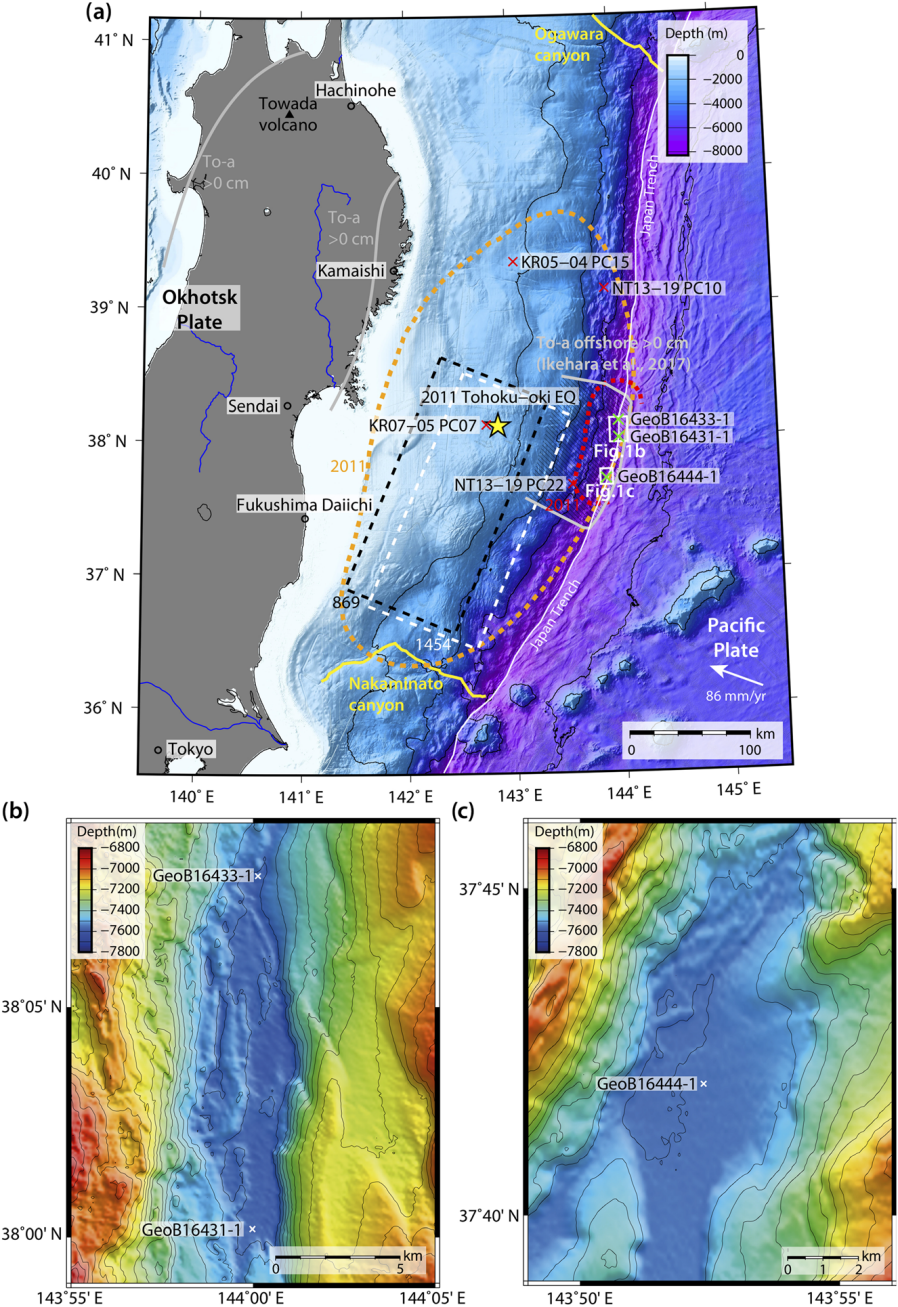
(a) Overview map of the Japan Trench, East of Honshu, showing the epicenter of the 2011 Tohoku‐oki earthquake (M_w_ 9; yellow star) with the 2 m (Sun et al., 2017) and 40 m (Chester et al., 2013) coseismic slip (dashed orange and red line, respectively); reconstructed rupture areas of both the AD 1454 M_w_ ≥ 8.4 Kyotoku earthquake (white dashed line, Sawai et al., 2015) and AD 869 M_w_ ≥ 8.6 Jogan earthquake (black dashed line, Sawai et al., 2012); the deposition area of the To‐a tephra (gray solid line offshore with shaded area, after Ikehara et al., 2017 and gray solid line onshore after Machida & Arai, 2003); Nakaminato and Ogawara canyon in the south and north, respectively (yellow dashed line); the core locations of the three trench cores GeoB16444‐1, GeoB16431‐1, and GeoB16433‐1 (green crosses) along the central Japan Trench (cruise SO219A, 2012), and four slope cores (red crosses, after Ikehara et al., 2013, 2017; Usami et al., 2018); (b) bathymetric map of the northern central basin (contour lines 50 m) with the core locations GeoB16431‐1 and GeoB16433‐1; (c) southern central basin (contour lines 50 m) with core location GeoB16444‐1.

The Japan Trench, located East of Honshu, Japan, is a convergent margin where the Pacific plate subducts in NW direction beneath the Okhotsk plate with average convergence rates of 80 to 86 mm/year (DeMets et al., 2010; Figure 1a). The trench axis has an N‐S to NNE‐SSW direction and has water depths of up to >8,000 m. The subducted oceanic crust has typical horst‐graben structures, resulting in several isolated trench basins along the trench axis (Nakamura et al., 2013; Figures 1b and 1c). The forearc slope can be divided into three sections, a gently dipping upper slope, a relatively steep middle slope with a mid‐slope terrace (only in the northern part of the Japan Trench and pinches out in the central part of it), and a lower slope (Arai et al., 2014; Kawamura et al., 2012). The lower slope has an angle of ~5° (Von Huene & Lallemand, 1990) and has been interpreted as a frontal accretionary prism (Nakamura et al., 2013). Several studies suggest that the Japan Trench is an erosive margin, where active forearc slope deformation influences the bathymetry (Arai et al., 2014; Boston et al., 2017; Von Huene & Lallemand, 1990). Only two canyon systems connect the shelf with the trench, the Nakaminato canyon in the south and the Ogawara canyon in the north (Figure 1a). This study focuses on two small trench basins in the central Japan Trench (Figures 1b and 1c), where no canyon system has direct influence (Kioka et al., 2019b).

The upper to middle slope sediment consists mainly of calcareous and diatomaceous silt to clay, due to high primary productivity, and a minor portion of dispersed volcanic and clastic grains (Arita & Kinoshita, 1984; Ikehara et al., 2016, 2013). The high primary productivity is linked to the interplay of the warm Kuroshio and Tsugaru currents and the cold Oyashio current (Ikehara et al., 2013, 2016). With water depths of <4,000 m and the occurrence of calcareous sediments, this environment lies above the carbonate compensation depth (CCD, Berger et al., 1976; Usami et al., 2018). Ikehara et al. (2013) report that hemipelagic sedimentation rates in the northern area vary between 12.7 and 41.1 cm/ka since the last glacial maximum (LGM), with an average of ~20 cm/ka excluding event deposits (core KR05‐04 PC15) or ~21–25 cm/ka including event deposits (core KR05‐04 PC15). In the central Japan Trench area, however, well‐dated tephra layers in core KR07‐05 PC07 indicate lower average sedimentation rates of ~4 cm/ka, including event deposits (Ikehara et al., 2013).

The lower slope and trench with water depths of >5,000 m, background (hemipelagic) sediments mainly consist of diatomaceous silty‐clay to clay (Ikehara et al., 2016, 2018), representing deposition below the CCD (Berger et al., 1976; Usami et al., 2018). On the lower slope, sedimentation rates including event deposits of ~15 cm/ka can be calculated based on bulk OC ^14^C ages (core NT13‐19 PC22; Ikehara et al., 2017). On the mid‐slope terrace, sedimentation rates including event deposits are higher, yielding ~95 cm/ka based on dated tephra layers (NT13‐19 PC10; Usami et al., 2018). In the central trench, the hemipelagic sedimentary succession with sedimentation rates of approximately 80–300 cm/ka (excluding event deposits) is episodically interrupted by event deposits (Bao et al., 2018; Ikehara et al., 2016, 2018; Kioka et al., 2019b). Sedimentation in the trench and on the midslope terrace is profoundly affected by recurrent strong earthquakes. For the M_w_ 9 2011 Tohoku‐oki earthquake, earthquake‐triggered event deposits (Ikehara et al., 2016, 2018; Kioka et al., 2019a, 2019b; McHugh et al., 2016; Noguchi et al., 2012; Oguri et al., 2013), trench collapses, and slumps (Kawamura et al., 2012; Nakamura et al., 2013; Strasser et al., 2013) have been observed.

Three fining‐upward event deposits (“thick turbidites” TT1–TT3) with thicknesses ranging from decimeters to meters have been described in several cores from the central Japan Trench (Ikehara et al., 2016, 2018). The bases of the fining‐upward units mainly consist of coarse silt to fine sand with parallel‐ and cross‐lamination and thicknesses in the range of centimeters to tens of centimeters. These coarse bases contain abundant quartz, volcanic glass shard fragments, pumice, lithic fragments (mainly composed of volcanic minerals), and feldspar (Strasser et al., 2017; Wefer et al., 2014). The finer‐grained sediments, towards the top of each fining‐upward unit, generally have higher diatom contents and are homogenous without evidence of bioturbation. These finer‐grained sections have thicknesses in the range of several decimeters to meters and are generally thicker than the coarser bases. In Unit TT3, the fine‐grained layers contain additionally calcareous nannofossils. The stratigraphic record dated by means of tephrochronology (Ikehara et al., 2016, 2017) allows correlating Units TT1–TT3 along the central Japan Trench to historic earthquakes (TT1: ad 2011 Tohoku‐oki Earthquake, TT2: ad 1454 Kyotoku Earthquake, TT3: ad 869 Jogan Earthquake; Ikehara et al., 2016, 2018).

To obtain the dating of sediments between tephra layers (Ikehara et al., 2016) at the hadal Japan Trench, which is lying far below the CCD, ^14^C ages were measured with high stratigraphic resolution on bulk OC retrieved from core GeoB16431‐1 (Bao et al., 2018; see Figure 1 for the location of coring site). Although bulk OC ^14^C ages do not represent the depositional age of the sediment due to the marine reservoir effect and reworked organic carbon, the results still show a clear linear relationship with depth for the hemipelagic background sediments, with a constant age offset of ~1,600 years as compared to the historical tephra layer (AD 915 Towada‐a [To‐a] tephra) and surface sediment (Bao et al., 2018; Ikehara et al., 2016; Kioka et al., 2019b). Within the event deposits TT2 and TT3, the bulk ages are additionally shifted by 3–6 ka indicating increased deposition of reworked organic matter (Bao et al., 2018).

Event deposits of the M_w_ 9 2011 Tohoku‐oki earthquake both on the midslope terrace and in the trench (unit TT1) are highly enriched in ^210^Pb and ^137^Cs (Ikehara et al., 2016; Kioka et al., 2019a; McHugh et al., 2016; Oguri et al., 2013). This finding indicates that they are composed of very young sediments, suggesting that surficial sediment remobilization is the dominant underlying process (Kioka et al., 2019a; McHugh et al., 2016). Due to the high OC content in surface sediments (Burdige, 2007), seismically triggered surface sediment remobilization has been further reported as an important process for carbon delivery into the hadal trench (Kioka et al., 2019a). A recent study (Molenaar et al., 2019) further provides direct stratigraphic evidence for the occurrence of surface sediment remobilization on the upper to middle slope of the northern Japan Trench. Abrupt shifts in ^210^Pb profiles on a young sedimentary record on the slope indicate three erosional events that were dated and correlated, respectively, with the three most significant earthquakes. Each of the three megathrust earthquakes of at least M_w_ 8 remobilized 4–12 cm of surface sediments, whereas smaller earthquakes did not cause detectable erosion at the slope site (Molenaar et al., 2019).

## 3 Approach, Materials, and Methods

Based on the investigation of XRF‐CS, MS, and density data, a statistically robust correlation of the stratigraphic record was established and compared with the existing correlation (Ikehara et al., 2016) made on visual core description and multisensor core logger (MSCL) measurements. The correlation was further validated by petrographic analysis of heavy grains from the sandy basal layers of targeted event deposits. Combined with ^14^C dates of bulk OC, the sediment remobilization processes are evaluated and discussed.

### 3.1 Materials

The three piston cores of this study, GeoB16431‐1, GeoB16433‐1, and GeoB16444‐1 were collected during the R/V Sonne fast response cruise SO219A in 2012, which was launched to detect the traces of the M_w_ 9 2011 Tohoku‐oki earthquake (Wefer et al., 2014). Cores GeoB16431‐1 (38° 00.177′N, 143° 59.981′E, 7,542 m water depth) and GeoB16433‐1 (38° 07.843′N, 144° 00.135′E, 7,525 m water depth) were collected in the northern central basin (Figure 1b) and core GeoB16444‐1 (37° 42.017′N, 143° 52.377′E, 7,529 m water depth) was retrieved in the southern central basin of the Japan Trench (Figure 1c). Both basins are approximately 40 km apart. These three cores were described and correlated to reveal the major earthquake history of the last 1,500 years (Ikehara et al., 2016).

### 3.2 XRF‐CS, MS, and Density

The three cores were analyzed with the Avaatech XRF core scanner at MARUM, University of Bremen. Continuous elemental variations were obtained at 1 cm scanning resolution with 20 s exposure time, 10 kV and 0.20 mA for Al, Si, S, K, Ca, Ti, Mn, Fe, as well as 30 kV and 0.90 mA for Br, Rb, Sr, Zr, respectively. The elemental intensities were transformed by using the centered‐log ratio (clr) transformation to avoid possible closed‐sum and asymmetry problems caused by down‐core physical and matrix variations (e.g., Lee et al., 2018; Peti et al., 2019; Weltje et al., 2015). The resulting data was further filtered by a Gaussian filter (*g*(*x*); Equation 1) to mitigate the effects of high‐frequency noise:

(1) $$g\left(x\right)\;=\;e^{\frac{-x^{2}}{2\;*1.83^{2}}},$$

where *x* is an array of 11 evenly spaced numbers from −5.5 to 5.5.

In addition, density and MS were measured with a sampling interval of 1 cm using the Geotek MSCL at MARUM, University of Bremen.

Multivariate statistical analysis was established using the 12 elements (Al, Si, S, K, Ca, Ti, Mn, Fe, Br, Rb, Sr, Zr) measured by the XRF‐CS combined with density and MS. To characterize the fingerprints of event deposits, we applied principal component (PC) analysis (PCA; Loska & Wiechuła, 2003) to reduce the original data dimensions (14 variables). The resulting PCs were further tested and grouped by Ward's Hierarchical clustering analysis (Ward, 1963) to perform the best group‐identification. Both PCA and Ward's Hierarchical clustering methods were performed using codes on the R platform and following the procedures outlined by Kassambara (2017a, 2017b).

### 3.3 Bulk Grain‐Size Analysis and Sediment Treatment

Bulk grain‐size analyses of selected samples from the sand bases TT2 and TT3 in core GeoB16431‐1, as well as TT3 in core GeoB16444‐1 (Table S1 in the supporting information) were performed after sonification (60 s, 10% of maximum intensity) using the Malvern Mastersizer 2000 at the University of Innsbruck.

The petrographic heavy grain analysis was conducted on two samples of TT2 in GeoB16431‐1 and TT3 in GeoB16444‐1 to assess turbidite‐internal variability. For comparison between basins and evaluation of intrabasinal variability, samples from individual turbidite (i.e., TT2 in GeoB16431‐1 and TT3 in GeoB16444‐1) were merged, and compared with another sample from TT3 in core GeoB16431‐1 (Table S1). Subsamples of all five sand samples were wet‐sieved (mesh width of 250, 125, and 63 μm). Using the very fine sand fraction (63–125 μm), we performed a heavy grain separation with sodium heteropolytungstates (ρ = 2.80 ± 0.02 g/cm^3^; LST Heavy Liquids). The separated heavy grains were embedded in epoxy resin, ground to expose the surface of the grains and finally polished using 0.25 μm diamond suspension.

### 3.4 EDX Analysis of Heavy Grains and Volcanic Glasses

Individual heavy grains were identified with energy‐dispersive X‐ray (EDX) analysis (Quantax, Bruker) coupled with a scanning electron microscope (JEOL JSM‐6010LV), whereas quantitative mineral, lithic fragment, and volcanic glass analyses were performed with an electron microprobe (JEOL 8100 Superprobe) at the University of Innsbruck. For each sample, about 100 grains were analyzed using the ribbon‐counting method (Mange & Maurer, 1992) and classified after Garzanti and Andò (2007), with an additional category of lithic fragments. The heavy lithic fragments were further examined for the presence of magnetic minerals and volcanic glasses (e.g., glass rim or glass matrix).

Quantitative chemical analysis of volcanic glasses included in the heavy lithic fragments was then performed with an electron microprobe (JEOL 8000 Superprobe) applying an acceleration voltage of 15 kV. A low beam current of 5 nA in raster mode (~8 × 6 to 12 × 9 μm) was used to minimize both element diffusion within glass and surface damage on glass. To maximize X‐ray count yields, Cl, S, and P were analyzed with a PETH crystal using a high‐intensity spectrometer. Measurement times for major and minor elements were 20 s for peaks and 10 s for backgrounds (20/10) of the respective Kα lines, despite S, P, and Cl were measured by 60/30 and Mn for 100/50.

### 3.5 Erosion Depth Modeling

Investigating the mechanism of the 3–6 ka shift in bulk OC ^14^C ages within event deposits compared to the hemipelagic background sediment (Bao et al., 2018; see more details in section 5.3 below) is essential to better understand remobilization processes and explain the observed event deposit sedimentary facies. We thus propose theoretical erosion depth models for remobilization processes on the slope and evaluate and compare with the results from the event deposits in the trench. The models are based on the principles of radioactive decay of ^14^C, evaluated against sedimentation rates in slope regions as the source areas of the event deposits. Here we assume that (1) the bulk OC analyzed for ^14^C within event deposits (Bao et al., 2018) represents the average value of perfectly mixed bulk OC, which was remobilized in the source area (slope) and along the pathway of the turbidity current, and (2) there is a constant bulk OC ^14^C age offset of 1.6 ka on the slope (see section 5.3 for validation and discussion about potential impacts of these assumptions on derived results).

From tephra‐stratigraphy and bulk OC ^14^C ages, variable sedimentation rates including turbidites on the upper and lower slope of the central Japan Trench have been reported between ~4.0 and ~95.0 cm/ka (Ikehara et al., 2013, 2017; Usami et al., 2018). However, the latter higher rates were reported from coring sites in small intraslope basins that are more influenced by deposition from sedimentary gravity flows. We excluded such depositional slope basins with higher sedimentation rates, because such slope basins are primarily sedimentary depocenters and not source area for sediment remobilization violating aforementioned assumption 1. In contrast, we consider sedimentation rates on the slope, where erosion can occur to be in the order of 4–20 cm/ka (as mentioned in section 2) for constructing a theoretical stratigraphic slope sequence with a linear age model. This age model is also consistent with calculated sedimentation rates of ~4 cm based on tephra‐stratigraphy on the seaward slope (Ikehara et al., 2017).

**Figure 2:**
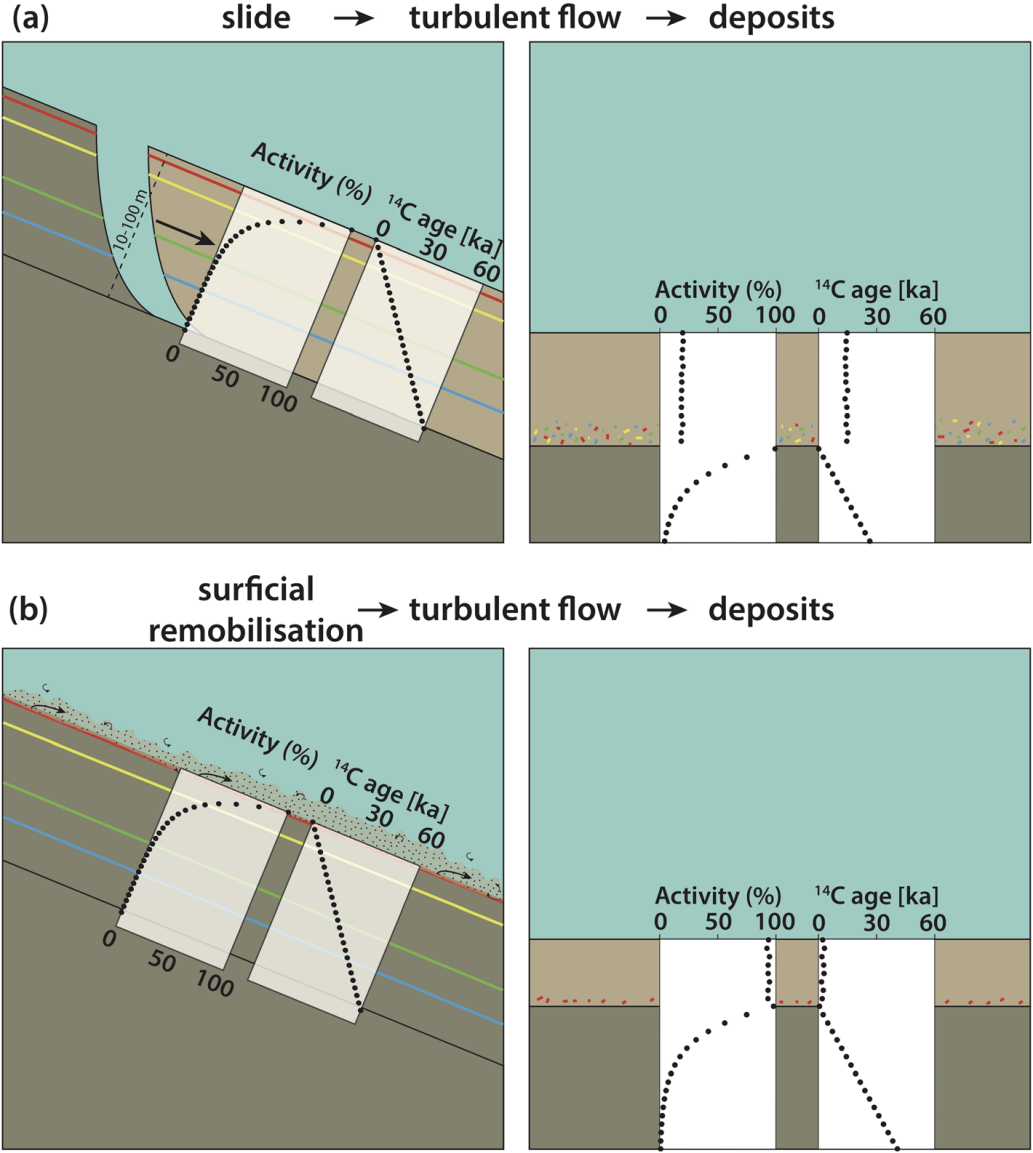
Simplified graphical representation of endmembers of the two remobilization models: (a) translational slide and (b) surficial sediment remobilization. For (a), the bulk OC ^14^C ages of event deposits are much older, because a higher amount of old carbon is remobilized. Note that, due to the exponential decay of ^14^C, the model is most sensitive when remobilizing young carbon, and less sensitive when remobilizing old or even blank carbon. In (b), the age shifts are expected to be small, due to remobilization of young surface sediment.

Conceptually, two different end member scenarios for remobilization can be considered (Figure 2). In a “translational slide scenario” (Figure 2a), a comparable thicker slope sequence (10–100 m thick deposited over several tens of thousands of years) is remobilized, mixed during transport (Assumption 1) and deposited as event deposits. The other end member scenario considers remobilization of only a thin veneer of young surficial sediment (Figure 2b; surficial remobilization). In the “translational slide scenario,” the ^14^C ages obtained from samples of the resulting mixed mass in the event deposits do not present a simple average age of eroded slope sequences. For the example as shown in Figure 2a, 30 ka would be the simple average age for a slope sequence that has been continuously deposited since 60 ka, but for the resulting mixed event deposit, average radiocarbon ages would be ~15 ka. This is because the radiocarbon age derived from the ^14^C activity decays exponentially over time. Thus, the contribution of younger sediment with higher ^14^C activity in the upper (younger) part of the slope sequence results in younger average radiocarbon age of the mixed (remobilized) sequence. On the other hand, when mixing a very thin slope sequence that was deposited over the last few hundred years (i.e., surficial sediment remobilization scenario, Figure 2b), the age shifts would be rather small.

By analyzing bulk OC ^14^C age shifts in event deposits (assumed to be mixed mass of the eroded sequence) and using the model framework presented in Figure 2, a theoretically eroded stratigraphic sequence can be inversely modeled. Here, we defined a linear age model consisting of 11 equally distributed age points (*t*_1_, *t*_2_,…,*t*_11_) over the theoretically eroded stratigraphic sequence. Thereby, *t*_1_ represents the surface age of 1.6 ka, whereas *t*_11_ represents the maximum age. For each of these linear age points *t*_*i*_ (*i* = 1, 2, …, 11), the corresponding ^14^C activity *A*_*i*_ at a given age point *t*_*i*_ can be calculated by solving the following equation:

(2) $$t_{i}\;=\;\frac{1}{\lambda }\;*\;\ln\left(\frac{A_{\text{in}}}{A_{i}}\right),$$

where *A*_*in*_ is the initial radiocarbon activity (100%), and λ is the decay constant of ^14^C (ln(2)/*τ*_1/2_ where *τ*_1/2_ is the half‐life of ^14^C (5,730 a)).

The average of all 11 activities (*A*_*i*_) represents the mixed activity or average activity (*A*_*av*_). The age range of the theoretical stratigraphic sequence has to be evaluated that their average activity (*A*_*av*_) corresponds to the ^14^C activity observed within the particular event deposit (*A*_*ed*_). The lowest ^14^C activity (*A*_11_) within this theoretically eroded stratigraphic sequence, representing the highest bulk OC ^14^C age (*t*_11_), also represents the oldest sediment incorporated in the mixing processes. By taking sedimentation rates into account (Ikehara et al., 2013, 2017), the oldest sediment of this linear theoretical sequence can then be used to estimate the maximum erosion depth (*z*).

A total OC (TOC) content decreasing over time directly influences the average activity (*A*_*av*_), because each age point (*t*_*i*_) is characterized by a different TOC content, which has to be considered in our models. Hence, we used weighted activity averages based on TOC content. The TOC content used for the models was estimated using data reported by Bao et al. (2018).

## 4 Results

### 4.1 MSCL and XRF‐CS Data

**Figure 3:**
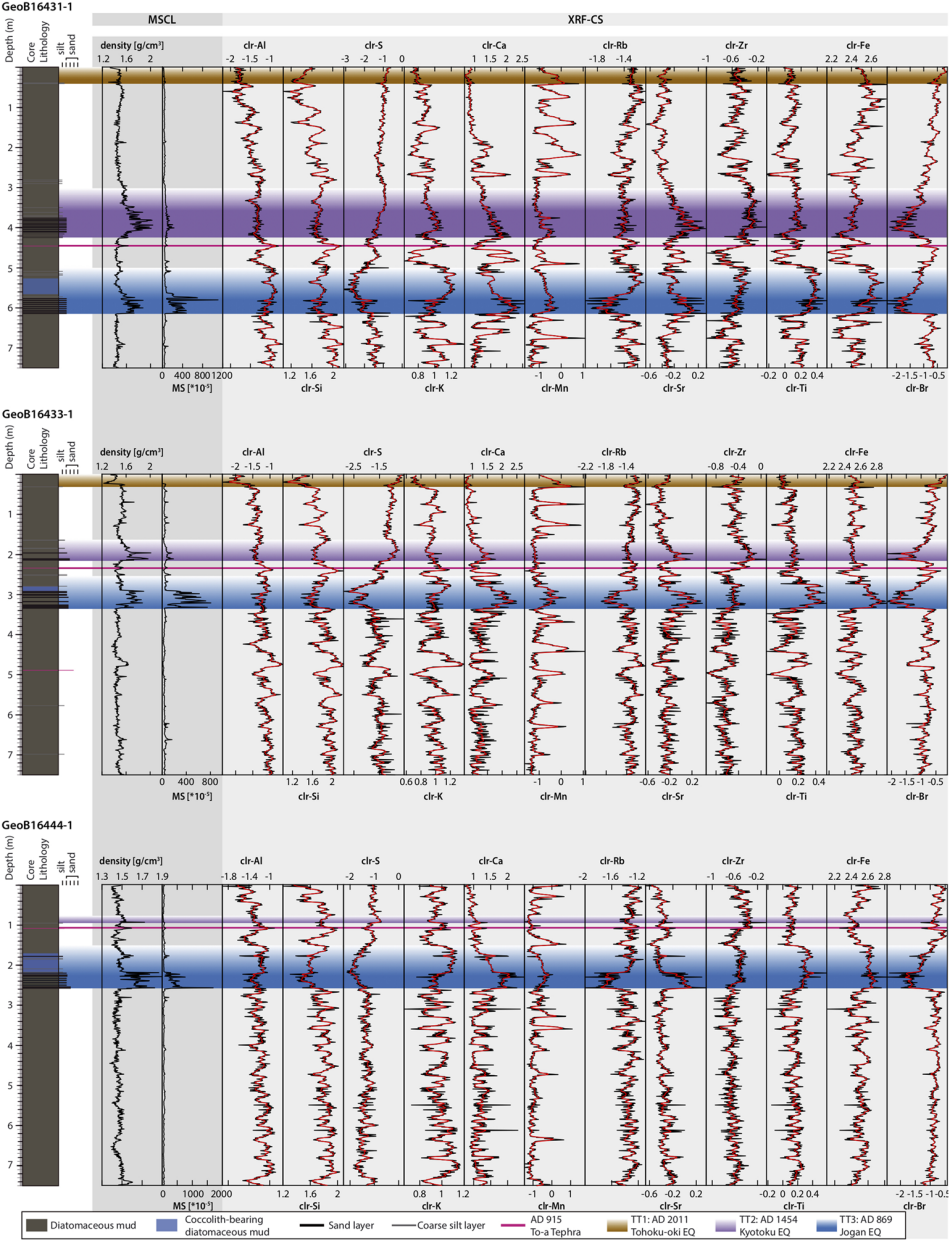
Lithology of cores GeoB16431‐1, GeoB16433‐1, and GeoB16444‐1 (Ikehara et al., 2016), with density, MS, and centered‐log ratio (clr) transformed XRF‐CS data (black), and the Gaussian filtering clr XRF‐CS data (red). The colored bands indicate the event deposits (TT1 = ad 2011 Tohoku‐oki Earthquake, brown; TT2 = ad 1454 Kyotoku Earthquake, purple; TT3 = ad 869 Jogan Earthquake, blue) and a tephra layer (ad 915 Towada [To‐a] tephra = pink; after Ikehara et al., 2016).

MSCL and XRF‐CS data (Figure 3) of cores GeoB16431‐1, GeoB16433‐1, and GeoB16444‐1 show high variations. The density shows typical values in the background sediment of 1.4–1.5 g/cm^3^ with increased values of 1.4–1.9 g/cm^3^ towards the sandy bases of TT2 and TT3 and slightly decreased values in TT1 (1.4 g/cm^3^). MS shows low values in the background sediment and TT1 (20 × 10^−5^ – 50 × 10^−5^), slightly increasing values in TT2 (~20 × 10^−5^ – 200 × 10^−5^), and high values in TT3 (~20 × 10^−5^ – 1,500 × 10^−5^). Mn shows a series of distinct peaks in the background sediment and TT1 but are rather stable within event deposits TT2 and TT3. Likewise, K shows peaks in the background sediment, while it is low (clr‐value <0.8) in TT1, intermediate in TT2 (0.9–1.0), and high (>1.0) in the fine‐grained top parts, especially, of unit TT3. Compositional differences between TT2 and TT3 can be further observed for several elements such as Ti and Fe. In TT2, both Ti (0.1–0.2) and Fe (2.4–2.5) show similar clr‐values as detected within the background sediment, whereas those in TT3 show significantly higher values (Ti: 0.2–0.5 and Fe: 2.6–2.8). Br shows clr‐values in the range of −1 to −0.5 in the background sediment without significant variations. However, it clearly shows decreased values in TT2 and TT3 with minimums of −2.0 in the sandy layers. In TT1 Br is slightly increased with values >−0.5. Si and Al values generally increase down‐core from approximately 1.4 to 2.0 and −1.8 to −1.0, respectively.

### 4.2 Multivariate Statistical Correlation

**Figure 4:**
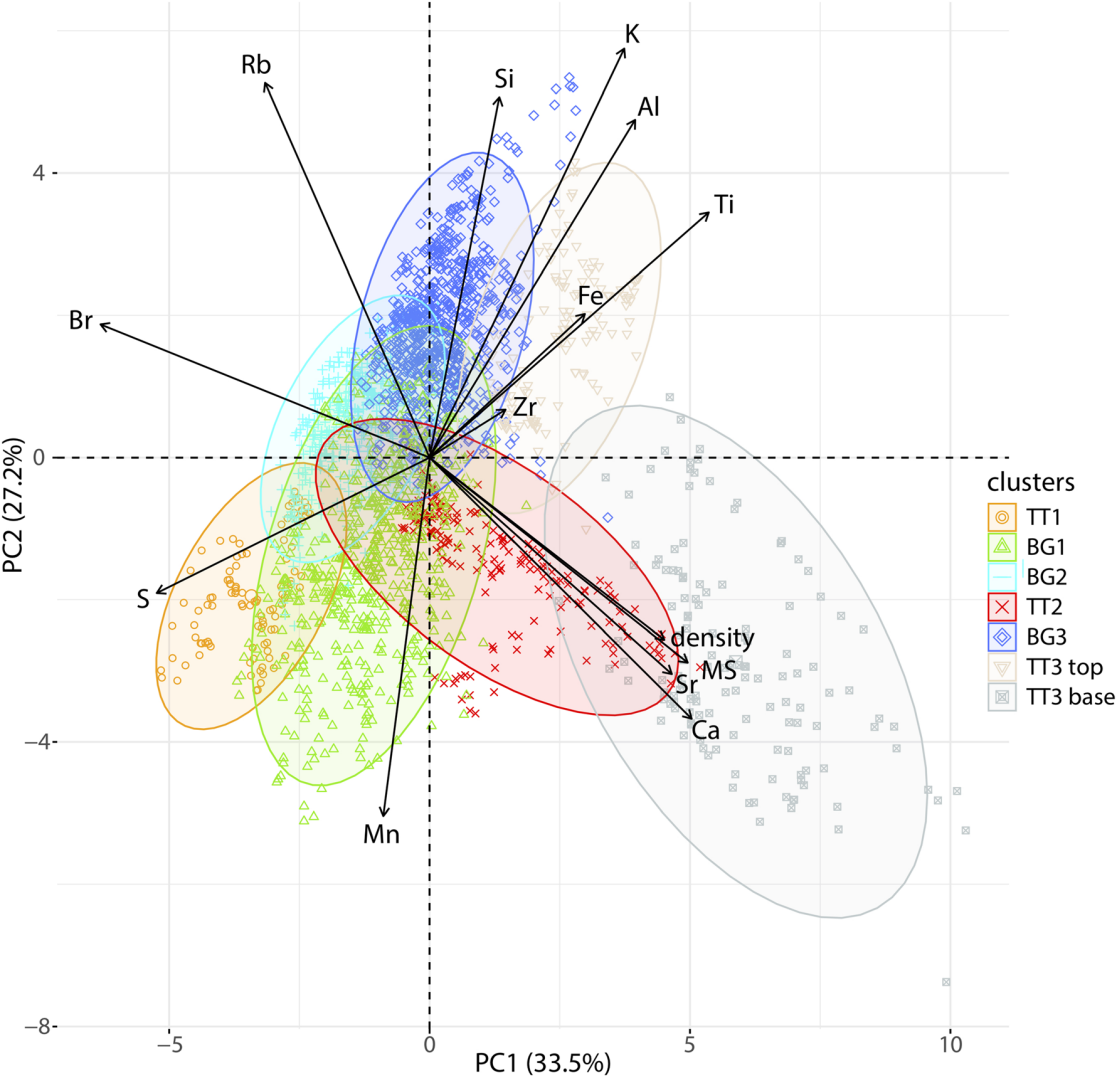
Biplot of PC1 (33.5%) and PC2 (27.2%), covering together >60% of the total data variation, colored in the seven clusters.

The data set presented in the previous section 4.1 was subjected to multivariate statistical analysis including PCA and cluster analysis. By applying PCA in a correlation matrix, ~95% of the information of the 14 variables (12 XRF‐CS elements, the density and the MS) are reduced to seven PCs. Based on Ward's Hierarchical clustering method, the seven PCs are grouped into seven clusters. The number of clusters is evaluated stepwise, starting with four as the four main lithological units (background sediment (BG) and TT1 to TT3; Ikehara et al., 2016) in order to test whether the correlation can be confirmed geochemically (see Figure S1 for the details). Initial results reinforce the notion that these main lithological units can mostly be reproduced independently by our multivariate statistical approach. However, as the detailed data variations can still be extracted and distinguished from the units of background sediments and TT3, respectively, we opt for seven clusters (Figure 4) which best reproduce the event‐stratigraphic correlation by Ikehara et al. (2016) and the additional detailed information provided by multivariate statistical approach.

The orange cluster (TT1) is highly associated with S and overlaps with the green cluster influenced by Mn. The red cluster (TT2) plots from the center towards density, MS, Sr, and Ca and lies on the opposite of Br. The dark‐gray cluster (TT3 base) also plots in the fields of density, MS, Sr, Ca, opposite of Br and partly overlaps with the red cluster. The light‐gray cluster (TT3 top) plots towards Ti, Fe, and Al. The green cluster (BG1) is mainly influenced by Mn, whereas the cyan cluster (BG2) is located in the center and is not dominated by a specific element. The blue cluster (BG3) is influenced by Si and K and is mainly associated with PC2.

### 4.3 Bulk Grain Size and Petrographic Analysis of Heavy Grains

**Figure 5:**
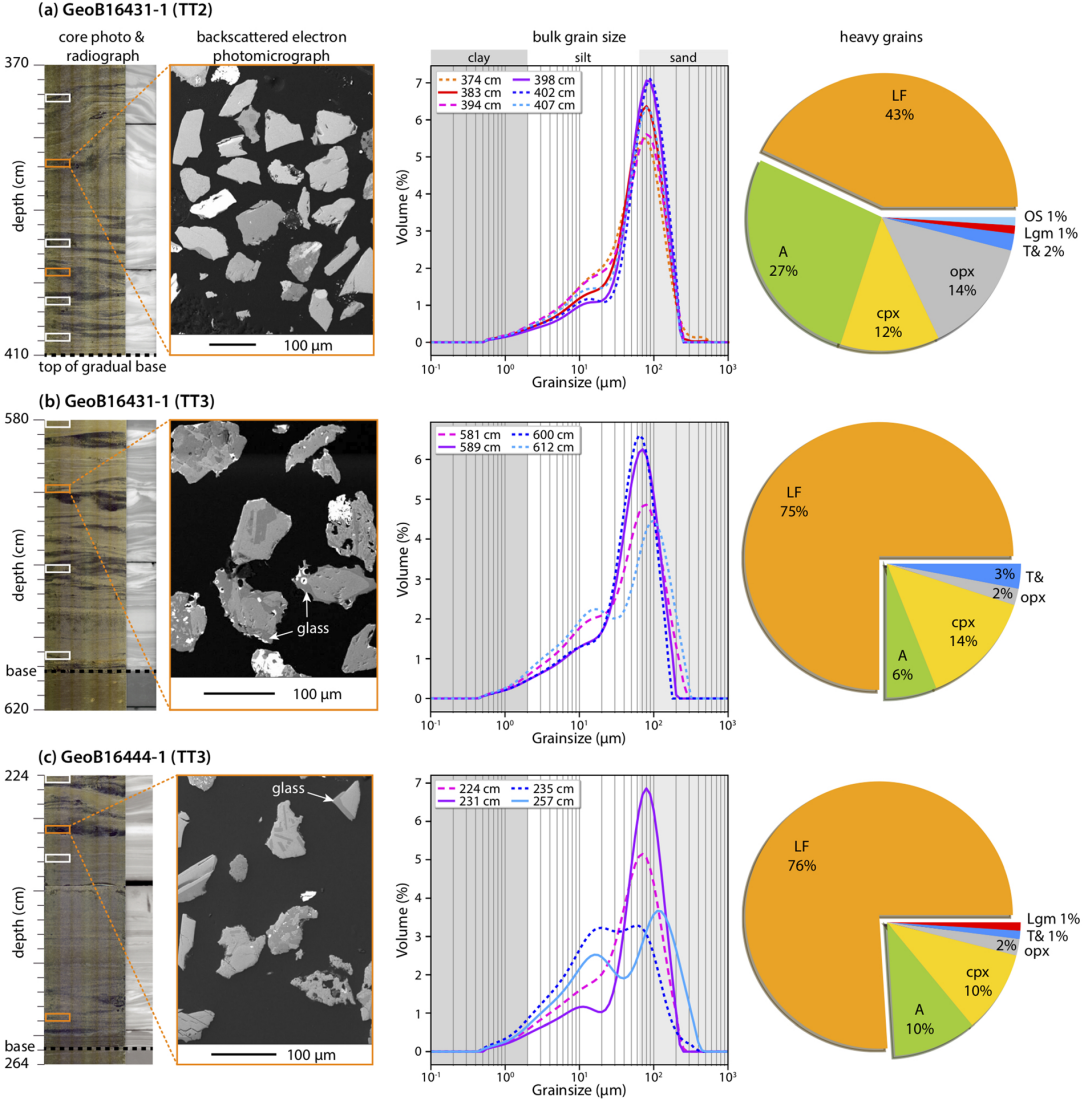
Core photos and backscattered electron photomicrographs of typical examples of heavy grains from sand bases of (a) GeoB16431‐1 TT2 (top of the gradual base at 410 cm), (b) GeoB16431‐1 TT3 (base at ~615 cm), and (c) GeoB16444‐1 TT3 (base at 262 cm). The photomicrographs show the variability of the angular to subangular heavy grains. White and orange rectangles in the core photos indicate the depth from which the sand samples were taken. Bulk grain size analysis yields left‐skewed distributions with peaks between 70 and 90 μm (warm colors at top to cold colors at bottom). The orange rectangles further mark samples used for petrographic analysis of heavy grains with corresponding proportions shown in the pie chart (LF = lithic fragments, a = amphiboles, cpx = clinopyroxene, opx = orthopyroxene, T& = titanium minerals and others, Lgm = low grade metamorphic minerals, OS = olivine and spinel). Note that in (a) and (c), two petrographic samples of each turbidite are lumped together to one representative pie chart as they are very similar, see Figure S2 for separate pie charts.

The bases of unit TT2 and TT3 are characterized by laminated to cross‐laminated very fine to fine‐grained sand. The bulk grain size distribution in the basal parts of TT2 and TT3 generally shows peaks varying between 70 and 90 μm (very fine sand) and minor amounts of silt to clay (Figure 5). The heavy grains from all five samples are angular to subangular (Figure 5). The bulk sand compositions are characterized by abundant quartz, feldspar, pumice, glass shard fragments, and lithic fragments (mainly volcano‐clasts; Wefer et al., 2014). Similar findings have been reported from a later cruise in 2016 (Strasser et al., 2017).

For the petrographic analysis of heavy grains, event deposit TT2 of core GeoB16431, represented by two samples, contains ~4 wt% heavy grains. Forty‐three percent of the 183 analyzed heavy grains are lithic fragments (LF) of mainly volcanic origin. Amphiboles (A), clinopyroxene (cpx), and orthopyroxene (opx) have share of 27%, 12% and 14%, respectively. Olivine and spinel (OS) and low‐grade metamorphic minerals (Lgm) contribute each with 1%, whereas titanium minerals and others (T&) contribute 2% (Figure 5). Furthermore, ~16% of the heavy grains include high MS minerals such as titanomagnetite and ~15% contain volcanic glass.

TT3 of core GeoB16431, represented by one sample, contains ~7 wt% heavy grains. For this sample, ~75% of the 98 heavy grains are LF of mostly volcanic origin. In addition, 14% cpx, 6% A, 2% opx, and 3% T& are present. High MS minerals constitute 27% and volcanic glasses 19% of the examined heavy grains.

TT3 of core GeoB16444, represented by two samples, contains ~7 wt% heavy grains. Of the 196 heavy grains, 76% are LF of mostly volcanic origin. In addition, 10% A, 10% cpx, 2% opx, 1% Lgm, and 1% T& are present. High MS grains compose 34% and volcanic glasses 35% of the examined heavy grains.

### 4.4 Glass Analysis

**Figure 6:**
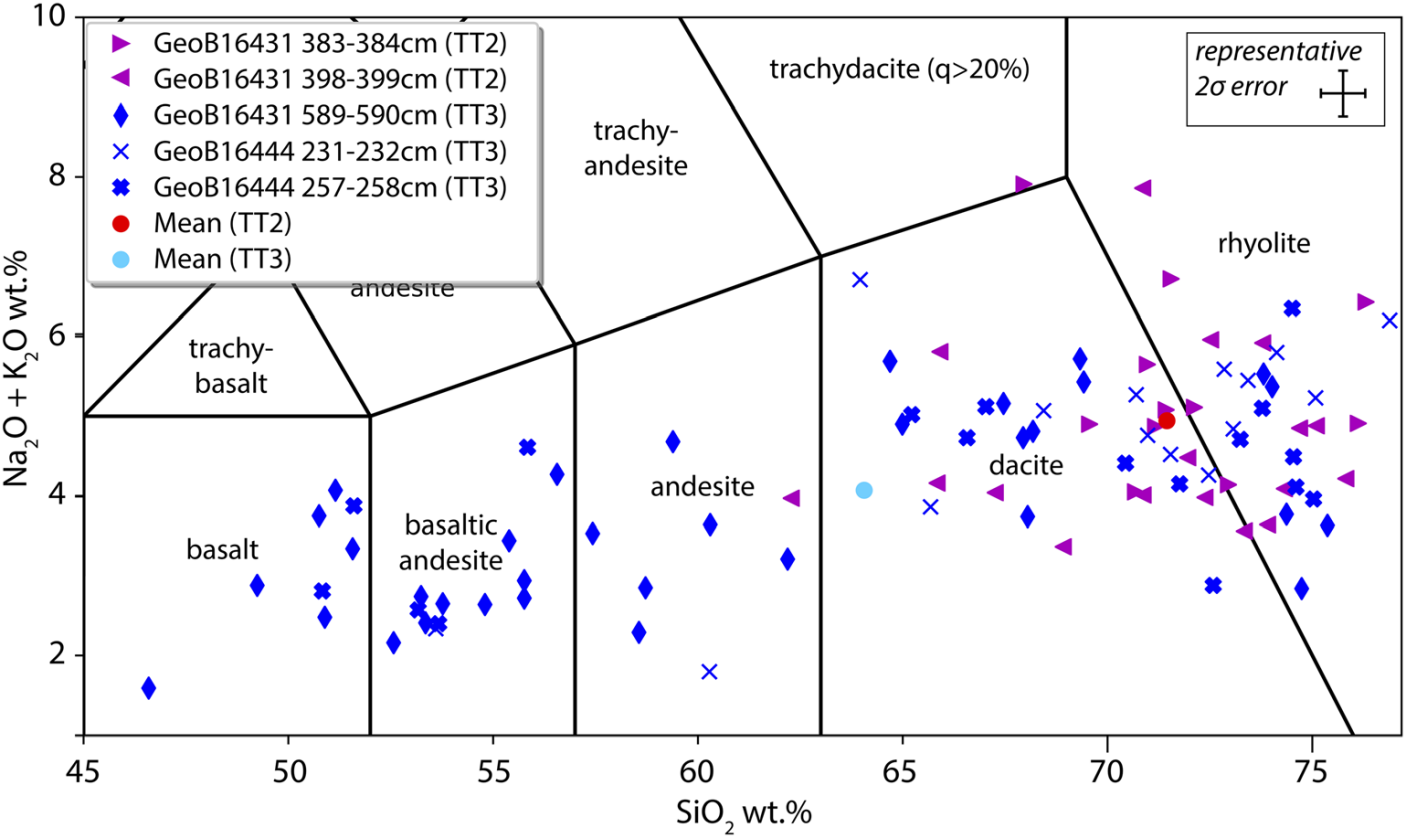
Total alkali‐silica (TAS) diagram showing the composition of volcanic glass found in the heavy grains of event deposits TT2 (violet symbols) and TT3 (blue symbols). The representative 2 sigma error refers to individual data points.

To compare the compositions of volcanic glass included in LF of the five heavy grain samples (section 4.4), their SiO_2_ and Na_2_O + K_2_O contents are plotted in a total alkali‐silica (TAS) diagram (Figure 6). Although the SiO_2_‐averages of glasses in TT2 and TT3 plot in the dacite field, their compositions are fairly diverse. Whereas glasses from TT3 show a wide range in SiO_2_ contents (mean SiO_2_ = 64.05 wt%, sd (standard deviation) = 8.93, *n* = 64) including several compositions in the fields of basaltic andesite and andesite, glasses from TT2 show a more restricted range in composition mostly plotting in the fields of dacite and rhyolite (mean SiO_2_ = 71.45 wt%, sd = 3.29, *n* = 28). The contents of Na_2_O + K_2_O within TT2 (mean Na_2_O + K_2_O wt% = 4.95, sd = 1.18, n = 28) are also slightly higher than in TT3 (mean Na_2_O + K_2_O wt% = 4.07, sd = 1.22, n = 64).

### 4.5 Compiled Multiproxy Data Characterizing Event Deposits and Background Sediment

**Figure 7:**
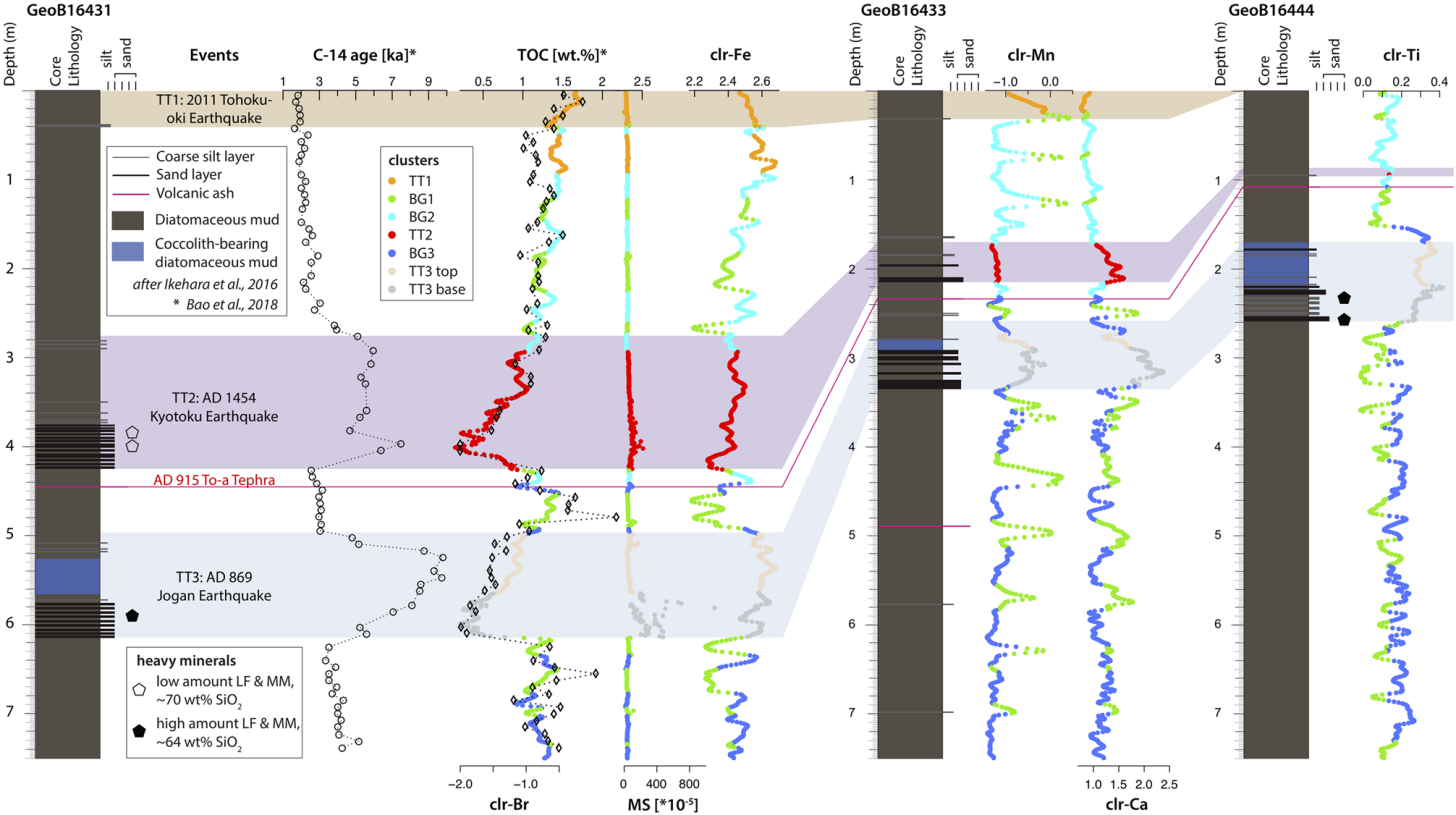
Core correlation of the central Japan Trench basins (after Ikehara et al., 2016) and the bulk OM ^14^C age distribution and TOC of GeoB16431 (Bao et al., 2018) supported by XRF‐CS, MS, and density data, colored in their specific clusters, and heavy mineral data (LF and MM = lithic fragments and minerals with high MS, respectively). Besides variabilities in thickness, grain size, TOC, and bulk OM ^14^C age distribution of the three event layers, there are well‐defined inherent chemical fingerprints (plotted in different colors of the associated clusters) which can be strikingly traced across the three cores from separated trench‐basins >40 km apart from each other (see Figure 1). Furthermore, the background sedimentation changes at the To‐a tephra from BG3 (dark blue) and BG1 (green) to BG1 (green) and BG2 (light blue).

In the following, we integrate our new data presented in sections 4.1, 4.4, with published data of lithological units (Ikehara et al., 2016), TOC, and bulk OC ^14^C (Bao et al., 2018), to comprehensively characterize the event‐stratigraphic sequence in the central Japan Trench by our new integrated multivariate statistical and multiproxy approach (Figure 7).

#### 4.5.1 Background Sediment (Diatomaceous, Partly Bioturbated Silt to Silty‐Clay)

The background sediment is represented by three clusters, BG1 (green), BG2 (light blue), and BG3 (dark blue), and shows a pattern‐change in all three cores at the AD 915 To‐a tephra. Below the To‐a tephra, the background sedimentary sequence is dominated by the alternations of the BG3 and the BG1 clusters, while the sequence above the tephra is dominated by the alterations between the BG1 and the BG2 clusters. The BG1 cluster is usually associated with Mn‐rich sediment layers, while the change from BG3 to BG2 across the To‐a tephra indicates a transition to relatively Si‐ and K‐poor overlying sediments (Figure 4). Ash layers, such as the To‐a tephra, are generally too thin (<1 cm) with respect to our sampling resolution and are embedded in the clusters of background sediment. The density of the background sediment typically varies between 1.4 and 1.5 g/cm^3^ and the MS between 20 × 10^−5^ and 50 × 10^−5^. TOC content varies between 0.88 and 2.17 wt% (average 1.27 wt%, sd = 0.25, *n* = 46). Bulk OC ^14^C ages show a strong linear relationship with depth, offset by approximately 1,600 years (Figure 7; Bao et al., 2018; Kioka et al., 2019b).

#### 4.5.2 TT1 (30–40 cm Thick Diatomaceous Clay With an ~0.5 cm Thick Sandy‐Silt to Silty Base, Overlaying a Thin [~2 cm] Reddish, Oxidized Layer)

The TT1 is mostly represented by the orange cluster in core GeoB16431‐1 and GeoB16433‐1 (Figure 7), but absent in GeoB16444‐1 due to the coring disturbance in the uppermost part of the gravity corer (Wefer et al., 2014). The cluster analysis does not uniquely depict the lower boundary of the TT1 as the orange cluster also occurs below the base of this event‐bed in core GeoB16431‐1 (Figure 7; see discussion below in section 5.1). For the XRF‐CS data, TT1 shows high S (≥ −1.0) and high Br (> −0.5, decreasing towards the base) compared to other units. Fe generally increases from 2.4 towards 2.6 downward, with a small peak of 2.7 to 2.8 right below the base at the reddish oxidized layer reported by Ikehara et al. (2016). Mn increases from −1.0 to >0.0 downward and has a distinct peak of up to 0.5 at the base (green cluster at the base of TT1 in GeoB16433; Figure 7). The MS is very similar to the background sediment (20 × 10^−5^ – 50 × 10^−5^), and the density (~1.4 g/cm^3^) is lower compared to the background sediment. The TOC content is high (1.20–1.75 wt%, average 1.49 wt%, sd = 0.17, *n* = 5). Besides the constant age offset of ~1.6 ka, the bulk OC ^14^C ages of TT1 do not show an additional age shift (Bao et al., 2019).

#### 4.5.3 TT2 (35–140 cm Thick Homogenous Diatomaceous Clays With a 2–50 cm Thick Very Fine Sand to Coarse Silt Base)

Unit TT2 is uniquely represented by cluster TT2 (red), characterized by high density (~1.5–2.0 g/cm^3^), slightly downward increased MS (~20 × 10^−5^ – 200 × 10^−5^) and downward increased Ca and Sr values (especially in the sandy base), and low Br with values ranging from −0.5 at the top to −2.0 in the sand bases. The sandy base in core GeoB16431‐1 (410–424 cm) is inversely graded and shows decreased Fe and Ti values of 2.3 and 0.0, respectively. For the entire overlaying graded unit, Fe and Ti have constant values of 2.4–2.5 and 0.1–0.2, respectively, and lie in the range of the background sediment. The heavy fraction of TT2 contains 43% lithic fragments of mainly volcanic origin, 27% amphibole and 14% orthopyroxene. The fraction of minerals with high MS (16%) is lower than that of TT3. Glasses found within the heavy fraction (e.g., glass rims of lithic fragments) are typically rich in SiO_2_ (mean SiO_2_ = 70.38 wt%, sd = 4.79, *n* = 34; Figure 6). In the inversely graded base (GeoB16431‐1 410–424 cm), density and MS increase gradually upward (Figure 3). Bulk OC ^14^C ages show (apart from the lowermost‐two samples in the basal sand layer) a uniform age shift of ~3.0–3.5 ka (Figure 7). TOC shows a downward decreasing trend and is generally lower than the background sediment (~0.20–1.20 wt%, average 0.9 wt%, sd = 0.37, *n* = 14; Bao et al., 2018).

#### 4.5.4 TT3 (65–115 cm Thick Homogenous Silty Clay and Coccolith‐Bearing Diatomaceous Mud Overlaying an ~40 cm Thick Basal Very Fine Sand Layer With a Sharp Erosive Base)

The sandy base of unit TT3 is represented by cluster “TT3 base” (dark gray) and the fine‐grained, muddy top is represented by cluster “TT3 top” (light gray; Figure 7). Cluster TT3 base is characterized by high density (~1.4–1.9 g/cm^3^), high MS (~20 × 10^−5^ – 1,500 × 10^−5^) and with downward increased Ca and Sr values higher than in TT2. Br ranges from −0.8 at the top to −2.0 at the bottom and is, thus, comparable with unit TT2. Fe and Ti are uniformly high with values of 2.6–2.8 and 0.2–0.5, respectively, compared to TT2 and the background sediment. The heavy fraction of TT3 comprises 75% lithic fragments of volcanic origin, 10–14% clinopyroxene, and 2% orthopyroxene. The content of high MS minerals (27–34%) in TT3 is higher than TT2. Volcanic glasses show a wide range of SiO_2_ contents covering mafic to felsic compositions (mean SiO_2_ = 64.05 wt%, sd = 8.93, *n* = 64; Figure 6). The TOC content of unit TT3 is low (0.22–0.80 wt%, average = 0.54 wt%, sd = 0.19, *n* = 12). The bulk OC ^14^C ages show a nearly constant age shift of ~6 ka in the thick fine‐grained upperpart and decrease to ~2 ka in the coarse‐grained base of the event deposit (Bao et al., 2018).

## 5 Discussion

In the following sections, we discuss the geochemical, physical (section 5.1), and petrographic (section 5.2) fingerprints of TT1–TT3 and their indication for spatial and stratigraphic provenance changes in the central Japan Trench. For spatial changes, we presume that the source area or sediment provenance could be geographically different, because different rupture areas generally affect respective margin segments. On the other hand, for stratigraphic provenance changes, we consider that the source area remains geographically unchanged, while that source sediment composition changes over time due to variable sediment input or different stratigraphic levels affected by the respective remobilization process (i.e., different erosion depth). In section 5.3, we present our theoretical model estimating erosion depths on the slope of each event deposits by using the ^14^C activities of the bulk OC reported by Bao et al. (2018). The inferred sediment remobilization processes induced by the three largest (>M_w_ 8.4) megathrust earthquakes in the Japan Trench over the last 1,500 years are then discussed.

### 5.1 Geochemical and Physical Fingerprints of the Event‐Deposit Stratigraphic Succession in the Central Japan Trench

The multivariate statistics (PCA and cluster analysis) integrating XRF‐CS, MS, and density data refine prior results based on visual and lithostratigraphic correlation by Ikehara et al. (2016) and provide new fingerprinting information for the event deposits. The statistical analysis, for the first time applied in submarine paleoseismology, not only allows to correlate event deposits across separated trench basins >40 km apart but also reveal distinctly different sediment sources remobilized by the ad 1454 Kyotoku (TT2) and the ad 869 Jogan (TT3) earthquakes (as mentioned below and in section 5.2).

The sandy bases of TT2 and especially TT3 are characterized by relatively high density and partly increased MS. The high values of MS within the base of TT3 can be explained by the high amount of high‐MS minerals, such as titanomagnetite (section 4.3). Likewise, both Fe and Ti, which are low in TT2 and high in TT3, indicating substantial concentrations of Fe and Ti phases within TT3, support this interpretation.

Also, Ca and Sr show increased values in the sandy bases of TT2 and TT3. This covariation can be caused by plagioclase, which is a common mineral in these layers (Wefer et al., 2014). However, the low Si counts do not fully support this hypothesis. Alternatively, the covariation of Ca and Sr is linked to the occurrence of biogenic calcite, such as foraminifera and coccolithophorids (Rothwell & Croudace, 2015). This finding suggests that at least parts of the source area of TT2 and especially TT3 is marine‐pelagic sediments located above the CCD, which corroborates the occurrence of coccolith‐bearing diatomaceous mud within TT3 in all the three studied cores (Ikehara et al., 2016). It is implied that the above‐mentioned chemical differences between both event deposits (TT2 and TT3) are not biased by grain size or TOC content, since both, grain‐size distribution (section 4.3) and TOC content (section 4.5, after Bao et al., 2018) of TT2 and TT3 are in the very similar range. In fact, we suggest that the XRF‐CS data corroborate the petrographic [of this study] and the sediment lithologic (Ikehara et al., 2016) observations.

The correlation of Br and TOC often relates to marine organic matter (Caley et al., 2011; Leri et al., 2010; McHugh et al., 2008; Ziegler et al., 2008). Hence, the overall substantial match (*R* ^2^ = 0.69) of Br and TOC (Figure 7) in core Geob16431‐1 suggests that TOC is mainly composed of marine organic matter. This is in line with previous observations based on molecular and stable carbon‐isotope analyses by Ishiwatari et al. (2000), who reported that the majority of organic matter in the Japan Trench is derived from marine plankton. Within Units TT2 and TT3, it is evident that grading influences the TOC content and Br values, which is interpreted to result from hydraulic sorting. Particulate organic matter is lighter than sand grains and, thus, preferentially found in the finer grain‐size fraction. Furthermore, organic matter content also has a strong relationship with mineral surface area and thus tends to be concentrated in fine‐grained sediments having high surface area (Mayer, 1994). Despite these two interpretations, however, the average TOC contents in both TT2 and TT3 are lower than in the directly surrounding background sediment. This implies that (1) a lower amount of organic material in the source sediment, (2) faster decomposition of remobilized suspended organic material in the water column before deposition in the event deposits, or (3) the organic matter remained in suspension and was transported further offshore.

The relatively high Br and high S values within cluster TT1 (AD 2011 event deposit (Ikehara et al., 2016)), are likely influenced by higher TOC and probably also higher sea water contents (cf., Huang et al., 2016; Rothwell & Croudace, 2015; Weltje et al., 2015), due to its recent and rapid deposition. Therefore, it is possible that the geochemical and physical signal of this shallow event deposit could change with further burial, leading to degradation of organic matter and compaction of the event deposit (TT1). For event deposits TT2 and TT3, however, the geochemical and physical fingerprints are not expected to change significantly, as discussed above.

Overall, our findings indicate that multivariate statistical approaches using XRF‐CS, MS, and density data can also be used as a fast and nondestructive tool in in submarine‐paleoseismological studies. It allows to quickly decipher geochemical and physical differences of event deposits and to improve event‐stratigraphic correlation in a fast and high‐resolution manner.

### 5.2 Inference of Source Areas for the Sandy‐Base of Event Deposits TT2 and TT3 Based on Petrographic Fingerprints of the Heavy Grains

The heavy fractions of the sandy bases of event deposits TT2 and TT3 in the central Japan Trench are dominated by lithic fragments of volcanic origin and volcanic minerals such as amphibole, clinopyroxene, and orthopyroxene (Figure 5). All these grains are generally angular to subangular (Figure 5), indicating a short subaqueous transport distance. While considering the absence of major sedimentary routing systems (e.g., canyons) for gravitational sediment transport from volcanic source regions to the central Japan Trench (Figure 1; Kioka et al., 2019b), fallout tephra on the trench slope are the most plausible sediment sources for the volcanoclastic grains within sandy bases of the event deposits triggered by the AD 1454 Kyotoku (TT2) and the AD 869 Jogan (TT3) earthquakes. Several of these tephra layers deposited in the study area can be directly linked to past eruptions (Aoki & Arai, 2000; Ikehara et al., 2016, 2017), such as the AD 915 eruption of the Towada volcano (To‐a tephra), the Haruna‐Futatsudake‐Ikaho eruption of Haruna Volcano in the sixth century (Hr‐FP tephra), and older eruption of the Towada Caldera over the last ~15 ka (e.g., To‐Cu tephra at ~6 ka).

For this spatially restricted area between ~37.0°N and 38.5°N of the central Japan Trench, volcanic ash originating from explosive volcanic eruptions and subsequent fallout tephra are not expected to vary spatially in terms of their geochemical and petrographical characteristics (Ikehara et al., 2017). The differences in heavy grain assemblages and SiO_2_ contents of volcanic glasses between the TT2 and TT3 (Figures 5 and 6) are thus interpreted to reflect remobilization of different tephra layers on the forearc slope. Moreover, the distinct difference of volcanic glass compositions (Figure 6) between TT2 and TT3 suggests that the sedimentary sequence remobilized during the AD 1454 Kyotoku‐earthquake comprised tephra from felsic volcanism, whereas the preceding AD 869 Jogan earthquake remobilized an older stratigraphic sequence comprising tephra from felsic to mafic eruptions.

Interestingly, the spatial distribution of the AD 915 To‐a tephra (Ikehara et al., 2017) overlaps the northern part of the reconstructed rupture area of the AD 1454 earthquake (as indicated in Figure 1a). The AD 915 eruption of the Towada volcano in the northern Tohoku area resulted from dacite‐rhyolite magmatic activity, for which whole‐rock SiO_2_ contents of eruptive rocks show values of ~70 wt% (Kudo, 2010). This value is strikingly similar to our SiO_2_ contents of volcanic glasses in the sandy base of TT2 (Figure 6). Therefore, the comparable SiO_2_ contents, together with the spatial congruence of the AD 915 marine tephra distribution and the AD 1454 earthquake rupture area, suggest that the AD 1454 earthquake triggered widespread remobilization of the 539 years earlier deposited To‐a tephra.

For the sand base of TT3, the broader spectrum of the glass compositions suggests (1) sediment remobilization of older tephra with more variable geochemical characteristics, (2) stratigraphically deeper erosion of a slope sequence that may have contained multiple tephra layers, or (3) higher sediment input from the upper slope, which possibly has more variable tephra distributions, due to shorter distance from land. We note that all three scenarios are consistent with the occurrences of tephra layers on the trench slope (Ikehara et al., 2017). These include the Hr‐FP tephra characterized by tholeiitic and calc‐alkaline rocks series with whole‐rock SiO_2_ contents between ~50 and 74 wt% (Japan Meteorological Agency, 2013; Soda, 1989; Usami et al., 2018) and older tephra from Towada Caldera, which evolved from andesitic–rhyolitic magmatic activities over the last ~15 ka (Kudo, 2010). As the cores used in this study are not long enough to reach these tephra layers in the depositional trench basin, further study is thus needed to reveal the complete remobilization history of TT3.

### 5.3 ^14^C as a Proxy for Erosion Depth of Sediment Remobilization by Strong Earthquakes

#### 5.3.1 Modeling Results

The ^14^C ages of bulk OC presented by Bao et al. (2018) show sediment‐unit‐dependent age variabilities. In the hemipelagic background sediment, bulk OC ^14^C ages show a strong linear relationship with depth, while the overall age offset is ~1.6 ka (Bao et al., 2018; Kioka et al., 2019b). Additionally, the unit TT1 triggered by the AD 2011 Tohoku‐oki earthquake does not show an additional bulk OC ^14^C age shift, indicating that no older OC was remobilized from the trench slope and deposited in the trench basin. This finding, as well as the increased TOC content within TT1, further supports the evidence of surficial sediment remobilization as the dominant mechanism behind TT1 (Burdige, 2007; Kioka et al., 2019a; McHugh et al., 2016; Molenaar et al., 2019).

The units TT2 and TT3, however, show low TOC and additionally 3 to 6 ka older bulk OC ^14^C ages compared to the overall 1.6 ka offset. Considering the general assumptions outlined in the method section 3.5, the older radiocarbon ages indicate that stratigraphically deeper (older) sediments were remobilized by the AD 1454 Kyotoku earthquake (TT2) and the AD 869 Jogan earthquake (TT3). An interpretation of remobilizing slightly deeper and older strata agrees well with the finding that the AD 1454 Kyotoku earthquake may have remobilized the To‐a tephra deposited in AD 915, not the earlier tephra layers (section 5.2).

Using Equation 2 solved for *A*_*i*_, we calculate erosion depths of a theoretical stratigraphic sequence on the slope required to be remobilized and mixed in order to explain the observed age shifts of event deposits in the trench basins (Figure 2, Table 1). For the model, it is assumed that the remobilization is equal at the top and the base of the eroded stratigraphic sequence (as it occurs in a conceptual translational slide, Figure 2a). The weighting of the ^14^C activities (*A*_*i*_) of the 11 equally distributed ages (*t*_1_ to *t*_11_, Table 1) over the theoretically eroded stratigraphic sequence is based on an assumed decreasing trend of TOC content with increasing ages, validated by the TOC data of Bao et al. (2018).

**Table 1:** Remobilization Models for (a) 3 ka Age Shift and (b) 6 ka Age Shift Based on Equation 2 Presented in Section 3.5 With a Linear Weighting Model Based on TOC Content

Theoretical stratigraphy	TOC [wt%]	(a) Linear bulk age (t_i_) [a BP]	(a) A_i_ (of ^14^C)	(b) Linear bulk age (t_i_) [a BP]	(b) A_i_ (of ^14^C)
t_1_	1.5	1,600	82.40	1,600	82.40
t_2_	1.4	2,440	74.44	3,560	65.01
t_3_	1.3	3,280	67.25	5,520	51.29
t_4_	1.2	4,120	60.75	7,480	40.46
t_5_	1.1	4,960	54.88	9,440	31.92
t_6_	1.0	5,800	49.58	11,400	25.18
t_7_	0.9	6,640	44.79	13,360	19.87
t_8_	0.8	7,480	40.46	15,320	15.67
t_9_	0.7	8,320	36.55	17,280	12.36
t_10_	0.6	9,160	33.02	19,240	9.75
t_11_ (= z)	0.5	10,000	29.83	21,200	7.70
Weighted average			A_av_ = 57.37		A_av_ = 39.90

*Note*. The linear bulk ages are chosen in the way that their individual activities result in a weighted average equal to the activity of the age shift (3 or 6 ka) plus the constant age offset of 1.6 ka.

An additional age shift of 3 ka (i.e., TT2) combined with the overall age offset of 1.6 ka results in a bulk OC ^14^C age (*t*_*ed*_) of ~4.6 ka (at the time of deposition), corresponding to a bulk OC ^14^C activity (*A*_*ed*_) of ~57.4% of the initial activity (*A*_*in*_) (after Equation 2). By evaluating the weighted average (*A*_*av*_) of the ^14^C activities of the 11 equally distributed ages of the theoretically eroded stratigraphic time interval (*t*_1_ to *t*_11_), it appears that sediments with ages of up to 10 ka were eroded, in order to yield a weighted average ^14^C activity (*A*_*av*_) in the range of 57.4% and to explain the 3‐ka age shift (Table 1). Considering the sedimentation rates on the slope of 4–20 cm/ka (Ikehara et al., 2013, 2017) and the constant age shift of 1.6 ka, ages of 10 ka can be estimated as corresponding to depths (z) of 33.6 cm (for 4 cm/ka) to 168.0 cm (for 20 cm/ka). By using different TOC contents in the model (Table S2), the maximum ages, which are required to be remobilized, correspond to erosion depths comparable to the initial model (28.8–144.0 cm with TOC contents of 1.5 to 1.0 wt%; 40.8–204.0 cm with TOC contents of 2.0–0.2 wt%; Table S2). This indicates that variable TOC contents do not significantly influence the model.

Likewise, for TT3, an additional age shift of ~6 ka together with the overall age offset of 1.6 ka results in a bulk OC ^14^C age (*t*_*ed*_) of ~7.6 ka (at the time of deposition), equivalent to an activity (*A*_*ed*_) of ~39.9%. By evaluating the weighted average (*A*_*av*_) of the ^14^C activities of the 11 equally distributed ages of the theoretically eroded stratigraphic time intervals (*t*_1_ to *t*_11_), it appears that erosion of sediments with OC ages of up to ~21.2 ka is required to obtain a weighted average activity (*A*_*av*_) in the above range that explain the 6‐ka age shift (Table 1). Considering the sedimentation rates on the slope of 4–20 cm/ka (Ikehara et al., 2013, 2017) and the constant age shift of 1.6 ka, ages of 21.2 ka can be estimated in depths of 78.4 cm (for 4 cm/ka) to 392.0 cm (for 20 cm/ka). By using different TOC contents (Table S3), the erosion depths do not change substantially (64.6–323.0 cm and 98.4–492.0 cm with TOC contents of 1.5–1.0 wt% and 2.0–0.2 wt%, respectively; Table S3).

Both models were further tested by using 88 equally distributed ages with TOC contents varying between 1.5 and 0.5 wt%, in order to increase the resolution of the stratigraphic eroded time interval and to obtain a more accurate mixed age. This change in the model, however, caused only small changes in erosion depth (31.6–158.0 cm for a 3 ka age shift; 70.8–354.0 cm for a 6 ka age shift).

#### 5.3.2 Limitation of the Modeling Results and Implication for the Dominant Sediment Remobilization Processes

Our erosion‐depth model can estimate erosion depth of earthquake‐triggered sediment remobilization, indicating if event deposits originate from thick (several tens of meters) submarine landslides or rather thin (dm to m range) translational slides and/or surficial sediment remobilization (cm to dm range).

The estimated thickness of the remobilized layer of the erosion depth model is based on the general assumptions outlined in section 3.5 and represent the maximum thickness required to explain the observed data. Based on Assumption 1, this includes the remobilization in the source area, as well as the remobilization along the pathway of the turbidity current. Strong turbidity currents transporting sand fraction (e.g., TT2 and TT3) have the potential to erode deeper into the stratigraphy on their pathway to the trench (Hunt, 2017), shaping small confined channel structures on the slope (e.g., as shown by Kawamura et al., 2012) and increasing the mixed bulk OC age of the final deposits. Such erosive, sandy turbidity currents can probably evolve from surficial sediment remobilization, if a significant sand fraction (e.g., shallow‐buried tephra layer) is incorporated (e.g., the ad 915 To‐a tephra in TT2; section 5.2). Hence, the actual remobilization thickness in the source area is expected to be lower, since coarse, sandy turbidity currents, such as TT2 and TT3, probably remobilized additional older carbon along their transport pathway. Finer grained turbidity currents, where no sand is incorporated (e.g., TT1), have only minor to no erosive power and thus the bulk OC age of their deposits represent the erosion depth in the source area.

Assumption 2 considers that the bulk OC ^14^C age offset of hemipelagic background sediment on the slope is ~1.6 ka. This assumption is justified due to the following reasonings: (1) event deposit TT1, which is interpreted as surficial sediment remobilization based on short‐lived radionuclides (Kioka et al., 2019a), does not show an additional radiocarbon age shift (i.e., same age as the hemipelagic background sediment; Bao et al., 2018); therefore, the bulk OC ^14^C age offset must be similar both in the slope as in the trench sediments (~1.6 ka). (2) If the slope sediment would have substantially younger ^14^C age, then TT1 would have remobilized deeper and older carbon, in order to have a bulk age of 1.6–1.8 ka (Bao et al., 2018). However, in that case, short‐lived radionuclides, such as excess ^210^Pb (Kioka et al., 2019a), which can only be detected in very young sediment (<150 a), would have been far below the detection limit.

By comparing our modeled remobilization depths in the range of decimeter to meter with reported remobilization depths of surficial sediment remobilization (1–9 cm, McHugh et al., 2016; 0.6–20.5 cm, Moernaut et al., 2017; and 4–12 cm, Molenaar et al., 2019), we conclude that surficial sediment remobilization cannot fully explain the TT2 and TT3 deposits. By comparing our modeled remobilization depths, however, to submarine translational slides, which often occur with thicknesses of several tens to even hundreds of meters (Hampton et al., 1996; Harders et al., 2010), it is evident that our modeled depths are much smaller, and therefore cannot be explained by large‐scale translational slides either. This is in line with the absence of large‐scale slide scars on the bathymetric data upslope of our studied basins. Occurrence of old bulk OC is thus expected to derive from (1) thin slides below the resolution of the bathymetric data, (2) deeper erosion of sandy turbidity currents due to incorporated tephra layers, and/or (3) lower‐than‐assumed sedimentation rates, especially on the steep lower slope, which is mainly influenced by hemipelagic deposition and remobilization during frequent earthquakes.

For event deposit TT2, we suggest that based on the evidence of the remobilized tephra layer of To‐a (as discussed in section 5.2) and the rather small age shift with small modeled erosion depths (minimum of 28.8 cm, maximum of 204.0 cm), the predominant supply process is widespread remobilization of young (surficial) sediment coupled with deeper erosion along the turbidity current pathway and potentially a minor contribution of locally landslide‐remobilized older carbon. Also, the possibility of deeper erosion by strong and sandy turbidity currents might result in increased bulk OC ^14^C ages. The low TOC content within TT2 can be explained by dilution from remobilized tephra and older sediment with low estimated TOC content.

For event deposit TT3, widespread remobilization of young sediment might still be a key process based on the relatively shallow modeled erosion depths (minimum of 64.6 cm, maximum of 492.0 cm, with a uniform age shift of 6 ka in resulting event deposits) and the absence of evident large‐scale slide scarps on the bathymetric data. The age of TT3, however, is not uniformly shifted by 6 ka. It decreases towards the base to 2 ka. Hence, our model shows rather maximum erosion depths for TT3. Overall, the age shift of TT3 can be explained by deeper erosion along the pathway of the erosive, sandy turbidity currents and a potential contribution of landslide‐remobilized older carbon with slide scarps below the bathymetric resolution.

### 5.4 Sediment Remobilization and its Erosion Depth: Role of Tephra Layers

This study indicates that remobilization of young sediment is an essential contributor for the sediment and OC delivery into the hadal trench. Also, our data indicate that erosion depths of sediment remobilization might vary based on the lithostratigraphic succession and the occurrence or absence of tephra layers in the source area. Sandy tephra layers, when shallowly buried and shaken by strong earthquakes for the first time, can be liquified (Harders et al., 2010; Moernaut et al., 2019) and thus enhance surficial sediment remobilization, for example, by facilitating very thin and shallow translational landsliding in the range of centimeter to decimeter thickness. Also, if a widespread, shallow‐buried tephra is incorporated in the remobilization process, sandy, erosive turbidity currents can evolve downslope, and erode in deeper and older strata on their pathway.

Event deposit TT2 of the AD 1454 Kyotoku earthquake is characterized by a sandy base, which is enriched in volcanoclastic grains interpreted to have originated from AD 915 To‐A tephra (section 5.2). During the strong seismic shaking for the first time, the tephra horizon may have liquified and triggered slightly deeper remobilization (i.e., deeper compared to the surficial sediment remobilization of the 2011 Tohoku‐oki earthquake, for which no tephra layer occurs in the shallow‐subsurface slope sequence). The incorporation of tephra in the mobilized surficial sediments would in turn form erosive turbidity currents, resulting in deeper erosion and subsequent deposition of the thick TT2 event deposits with increased ^14^C age and with a sandy base mainly consisting of volcanoclastic materials.

Similar features can also be observed from event deposit TT3. The Hr‐FP eruption distributed tephra over parts of the study area, ~310 to 331 years before the AD 869 Jogan earthquake (Okuno et al., 2019). This tephra did not experience strong earthquake shaking prior to the AD 869 Jogan earthquake, and thus may have been liquified during the strong shaking, leading to very thin, surficial translational landslides evolving into erosive, sandy turbidity currents. The sandy turbidity currents, in turn, would have increased the erosion downslope and result in older bulk OC ^14^C age shifts. Older tephra layers were likely either already remobilized by prior earthquakes, possibly during the second to third Century AD, or 3.8 to 4.1 ka ago (Kioka et al., 2019b; Usami et al., 2018), or experienced seismic strengthening (Moernaut et al., 2019). Such seismic‐strengthened tephra layers may still have facilitated the initiation of translational slides in the sediments directly overlying the tephra (Wiemer et al., 2015). These slides could be in the thickness range of decimeter to meter and also contribute to the delivery of old carbon into the hadal trench. The strengthened tephra may not have been entrained during remobilization (e.g., Wiemer et al., 2015), and thus, the petrographic fingerprint of the tephra in the translational slides remains similar to the surficial sediment remobilized tephra (i.e., to the very thin and shallow translational landsliding in the range of cm to dm).

Considering the TT1 and the Tohoku earthquake, there was no significant tephra layer deposited during the last 11 centuries before this event. The last thick and widely distributed tephra (To‐a) may have been mostly remobilized by the preceding 1454 Kyotoku earthquake. Thus, surficial sediment remobilization during the 2011 earthquake was controlled by the earthquake shaking parameters and the physical properties of the surface sediments without the influence of tephra layers in the slope sequence. Consequently, surficial sediment remobilization only eroded a few cm of slope sediments (e.g., 1–9 cm, McHugh et al., 2016; 4–12 cm Molenaar et al., 2019). The resulting fine‐grained turbidity currents were nonerosive, since no sand/tephra was incorporated, and the TOC content of the final event deposit is elevated, due to remobilization of only the organic‐rich surface sediments (Burdige, 2007; Kioka et al., 2019a). Such resulting muddy event deposits can be challenging to be detected in the stratigraphic record, since distinct sandy bases are lacking, and the increased TOC content may decompose over time.

This study, together with the observation reported by Kioka et al. (2019a), concludes that surficial sediment remobilization is an important process to initiate the sediment transfer into the hadal trench. Erosion behaviors on the trench slope as well as the depositional signatures (volume, geochemical, physical, and petrographic fingerprints) of resulting event deposits in the terminal basins, are primarily controlled by the stratigraphy of the slope sequence, that is, mainly whether or not it contains recently deposited tephra. A similar process has been observed on the composition of turbidites from the Cascadia margin where a widespread major tephra layer (Mazama ash) was mainly remobilized during the first strong earthquakes (Goldfinger, 2009; Goldfinger et al., 2008).

Event deposits initiated by surficial sediment remobilization have been proposed to be a promising tool for paleoseismology as they likely provide complete earthquake records. Compared to translational slope failures, neither a long sediment recharge time in the source area nor deep “weak” layers (e.g., sensitive clay), or porewater overpressure, are required to remobilize surficial slope sediment. Therefore, it is important to reconstruct remobilization processes as far back in time as possible. We propose that the evaluation of bulk OC ^14^C age shifts within event deposits can help to assess predominant remobilization processes over the last ~30 ka, within the radiocarbon time span.

Moreover, in some settings, the volume of such event deposits may link to earthquake parameters such as seismic intensity (Moernaut et al., 2017), while the amount of coarse pulses in event deposits may provide only a crude record of the earthquake source time function (Goldfinger et al., 2007; Patton et al., 2015). We document in our study that the link of event deposits to primary earthquake parameters may only be valid if considering the presence of shallow subsurface tephra within the source slope sequences and its behavior upon strong seismic shaking. Shallow‐buried tephra can easily be remobilized and affect downslope erosion, as well as the volume, and the geochemical and physical fingerprint of the resulting event deposit.

## 6 Conclusions

We have applied multivariate statistics on XRF‐CS, MS, and density data, for the first time in submarine paleoseismology, to investigate the geochemical, physical, and petrographic fingerprints of earthquake‐triggered event deposits in the central Japan Trench. Together with bulk OC ^14^C age data and the related erosion model, we have evaluated the predominant remobilization processes and reached the following conclusions:

1. Characteristic sediment properties of the three different event deposits TT1, TT2, and TT3 in the central Japan Trench were established, improving the event stratigraphic correlation among core locations in two basins >40 km apart.
2. Initial surficial sediment remobilization partly coupled with deeper erosion downslope is found to be a significant process for earthquake‐triggered sediment remobilization. The thickness of remobilized slope strata, as well as the erosion along the pathway of the turbidity current, can vary, if tephra layers are incorporated in such remobilization process.
3. Our results support the hypothesis that event deposit TT1 was produced by earthquake‐triggered remobilization of surficial slope sediments during the AD 2011 Tohoku‐oki earthquake. For the units TT2 (AD 1454 Kyotoku earthquake) and TT3 (AD 869 Jogan earthquake), we suggest surficial sediment remobilization as the predominant remobilization process, augmented by additional remobilization and deposition of old OC. Important amounts of old OC in the event deposits can be provided by (1) the strong, sandy, and erosive turbidity currents incorporating older carbon along its pathway, and in part (2) deep erosion of older strata down to mechanically weak, seismic‐strengthened tephra layers.
4. Our data indicate that the presence of shallow‐buried tephra in the slope stratigraphy during earthquake‐shaking strongly influence the erosion depth in the source area and on the pathway of transport, as well as the geochemical, physical, and petrographic fingerprints of the resulting event‐deposits. The link from event deposits to primary earthquake parameters may thus only be valid if the shallow‐subsurface tephra on the source slope is considered.
5. Our approach potentially allows to constraining sediment remobilization processes over the last ~30 ka, within the radiocarbon time span, and better assessing whether the event‐stratigraphic record represents a complete and continuous earthquake record.

## Supporting information

Supporting Information S1

## Data Availability

The bathymetric data used in Figure 1 are available at JAMSTEC‐DARWIN database (http://www.godac.jamstec.go.jp/darwin/e) and Bundesamt für Seeschifffahrt und Hydrographie (https://www.bsh.de/DE/DATEN/Ozeanographisches_Datenzentrum/Vermessungsdaten/Nordpazifischer_Ozean/nordpazifik_node.html). XRF‐CS and MSCL data are available on PANGAEA (https://doi.pangaea.de/10.1594/PANGAEA.916028).
