# Supplementary material for: Multivariate Statistical and Multiproxy Constraints on Earthquake‐Triggered Sediment Remobilization Processes in the Central Japan Trench
Source: Geochem Geophys Geosyst. 2020 Jun 18;21(6):e2019GC008861. doi: 10.1029/2019GC008861 (PMC7379269; doi:10.1029/2019GC008861)
Supplement: Supplementary file 1 — Supporting Information S1 [file GGGE-21-e2019GC008861-s001.pdf]

[Geochemistry, Geophysics, Geosystems]

Supporting Information for

[Multivariate statistical and multi-proxy constraints on earthquake-triggered sediment remobilization processes in the central Japan Trench]

T. Schwestermann[1], J. Huang[1], J. Konzett[2], A. Kioka[1,3], G. Wefer[4], K. Ikehara[5], J. Moernaut[1], T.I. Eglinton[6], M. Strasser[1,4]

1. Institute of Geology, University of Innsbruck, Innrain 52, 6020 Innsbruck, Austria
2. Institute of Mineralogy and Petrography, University of Innsbruck, Innrain 52, 6020 Innsbruck, Austria
3. Department of Earth Resources Engineering, Kyushu University, Nishi-ku, Fukuoka 819-0395, Japan
4. MARUM – Center for Marine Environmental Sciences, University of Bremen, Leobener Str. 8, 28359 Bremen, Germany
5. Geological Survey of Japan, National Institute of Advanced Industrial Science and Technology (AIST), Tsukuba Central 7, 1-1-1 Higashi, Tsukuba 305-8567, Japan
6. Geological Institute, ETH Zürich, Sonneggstrasse 5, 8092 Zürich, Switzerland

Contents of this file

Figure S1
Figure S2
Table S1
Table S2
Table S3

Introduction

The supporting information provides additional figures concerning the number of clusters used for our cluster analysis (Figure S1) and pie charts of all five heavy grain samples, respectively (Figure S2). Detailed information about sample depths for bulk grain size and heavy grain analysis is presented in table S1. Also, additional weighting models used for the erosion depth evaluation of the 3-ka age shift (Table S2) and the 6-ka age shift (Table S3) are presented.

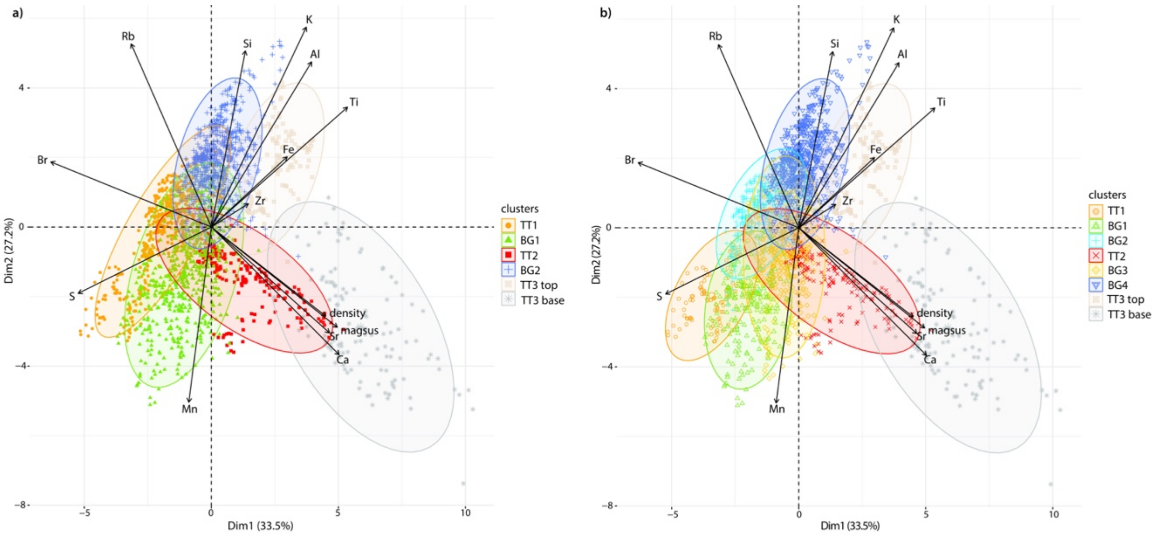

Figure S1. a) Biplot of 7 PCs with 6 clusters, b) biplot of 7 PCs with 8 clusters. The statistical patterns of event deposits do not change significantly from 6 to 8 clusters. In the 6-cluster model, the light blue cluster (BG2) is merged into the orange cluster (TT1), suggesting that the 6-cluster model is inadequate to fully reproduce the event-stratigraphic sequence. The 8-cluster model is splitting up the green cluster (BG1) in two sub-clusters, which does not add additional information for this study. Therefore, we conclude 7 clusters are the optimal cluster model (Fig. 4).

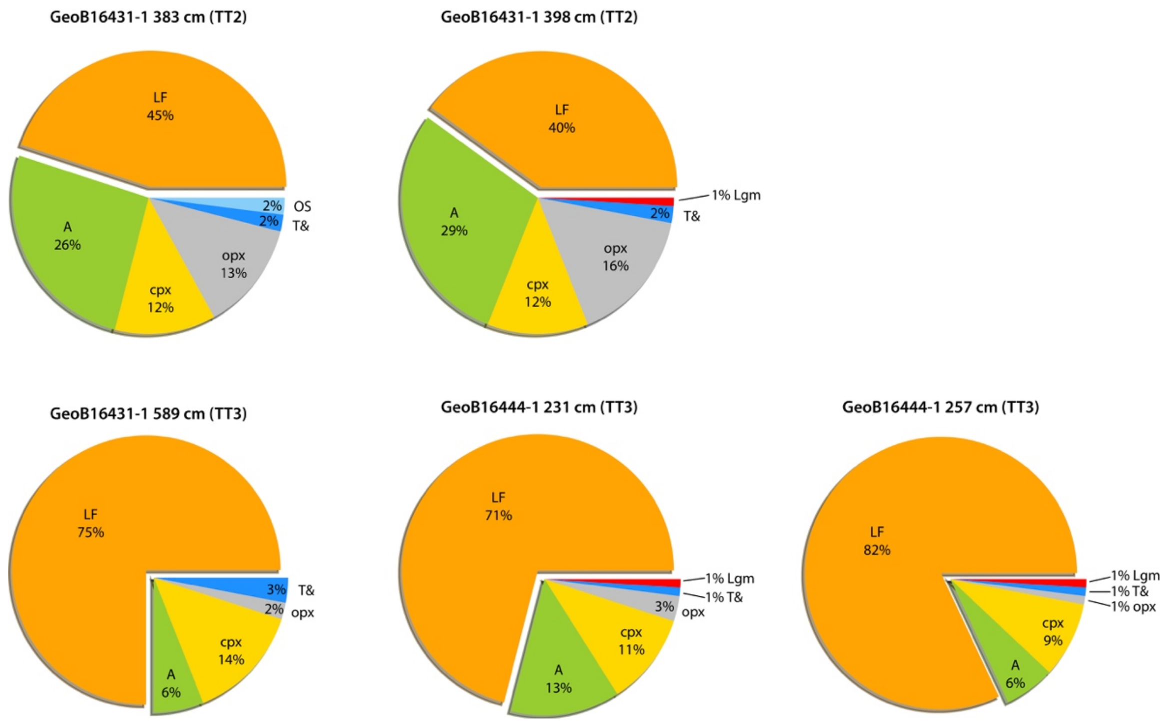

Figure S2. Pie charts of all five heavy grain analyses, respectively. The results show that internal variabilities of the studied turbidites are rather small.

Table S1. List of sample depths for bulk grain size and heavy grain analyses

Core	event	sample depth (cm)	bulk grain size	heavy grain
GeoB16431-1	TT2	374-375	x	
GeoB16431-1	TT2	383-384	x	x
GeoB16431-1	TT2	394-395	x	
GeoB16431-1	TT2	398-399	x	x
GeoB16431-1	TT2	402-403	x	
GeoB16431-1	TT2	407-408	x	
GeoB16431-1	TT3	590-581	x	
GeoB16431-1	TT3	589-590	x	x
GeoB16431-1	TT3	600-601	x	
GeoB16431-1	TT3	612-613	x	
GeoB16444-1	TT3	224-225	x	
GeoB16444-1	TT3	231-232	x	x
GeoB16444-1	TT3	235-236	x	
GeoB16444-1	TT3	257-258	x	x

Table S2. Remobilization model for a 3 ka age shift (A_{av} ~57.4) with linear weighting (TOC content) of a) 1.5 to 1.0 wt% and b) 2.0 to 0.2 wt%.

Theoretical stratigraphy	a) TOC [wt%]	a) Linear bulk age (t_i) [a BP]	a) A_i (of ^{14}C)	b) TOC [wt%]	b) Linear bulk age (t_i) [a BP]	b) A_i (of ^{14}C)
t_1	1.50	1600	82.40	2.00	1600	82.40
t_2	1.45	2320	75.53	1.82	2620	72.84
t_3	1.40	3040	69.23	1.64	3640	64.38
t_4	1.35	3760	63.45	1.46	4660	56.91
t_5	1.30	4480	58.16	1.28	5680	50.30
t_6	1.25	5200	53.31	1.10	6700	44.46
t_7	1.20	5920	48.86	0.92	7720	39.30
t_8	1.15	6640	44.79	0.74	8740	34.74
t_9	1.10	7360	41.05	0.56	9760	30.71
t_{10}	1.05	8080	37.63	0.38	10780	27.14
t_{11} (= z)	1.00	8800	34.49	0.20	11800	23.99
weighted average			**57.25**			**57.32**

Table S3. Remobilization model for a 6 ka age shift (A_{av} ~39.9) with linear weighting (TOC content) of a) 1.5 to 1.0 wt% and b) 2.0 to 0.2 wt%.

Theoretical stratigraphy	a) TOC [wt%]	a) Linear bulk age (t_i) [a BP]	a) A_i (of ^{14}C)	b) TOC [wt%]	b) Linear bulk age (t_i) [a BP]	b) A_i (of ^{14}C)
t_1	1.50	1600	82.40	2.00	1600	82.40
t_2	1.45	3220	67.74	1.82	4040	61.34
t_3	1.40	4840	55.68	1.64	6480	45.66
t_4	1.35	6460	45.77	1.46	8920	33.99
t_5	1.30	8080	37.63	1.28	11360	25.30
t_6	1.25	9700	30.93	1.10	13800	18.84
t_7	1.20	11320	25.43	0.92	16240	14.02
t_8	1.15	12940	20.90	0.74	18680	10.44
t_9	1.10	14560	17.18	0.56	21120	7.77
t_{10}	1.05	16180	14.12	0.38	23560	5.78
t_{11} (= z)	1.00	17800	11.61	0.20	26000	4.31
weighted average			**39.93**			**39.84**
